# Progress in Luminescent Materials Based on Europium(III) Complexes of β-Diketones and Organic Carboxylic Acids

**DOI:** 10.3390/molecules30061342

**Published:** 2025-03-17

**Authors:** Qianting Chen, Jie Zhang, Quanfeng Ye, Shanqi Qin, Lingyi Li, Mingyu Teng, Wai-Yeung Wong

**Affiliations:** 1 Department of Chemistry, School of Pharmaceutical and Chemical Engineering, Taizhou University, Taizhou 318000, Zhejiang Province, China; 2 Faculty of Chemistry and Chemical Engineering, Yunnan Normal University, Kunming 650500, China; 3 Department of Applied Biology and Chemical Technology and Research Institute for Smart Energy, The Hong Kong Polytechnic University, Hung Hom, Hong Kong, China

**Keywords:** rare earth luminescent materials, europium complexes, β-diketones, organic carboxylic acids

## Abstract

Europium(III) β-diketone and organic carboxylic acid complexes are designable, easy to prepare, and easy to modify and have excellent fluorescence properties (narrow emission spectral band, high colour purity, long fluorescence lifetime, high quantum yield, and a spectral emission range covering both the visible and near-infrared regions). These complexes play important roles in popular fields such as laser and fibre-optic communications, medical diagnostics, immunoassays, fluorescent lasers, sensors, anticounterfeiting, and organic light-emitting diodes (OLEDs). In the field of light-emitting materials, europium complexes are especially widely used in OLED lamps, especially because of their high-efficiency emission of red (among the three primary colours); accordingly, these complexes can be mixed with blue and green phosphors to obtain high-efficiency white phosphors that can be excited by near-ultraviolet light. This paper reviews the red-light-emitting europium complexes with β-diketone and organic carboxylic acid as ligands that have been studied over the last five years, describes the current problems, and discusses their future application prospects.

## 1. Introduction

In 1869, only six rare earth elements (REEs) had been discovered, and their existence challenged Mendeleev’s theory of the periodicity of the elements. By the late 19th century, new REEs had been proposed, but it was not until 1907 that all of the rare earth elements were finally isolated. After 1913, with the development of Bohr’s atomic model and Moseley’s X-ray spectroscopic studies, the position of the F-block in the periodic table was finally rationalized [1]. The REEs we now refer to include lanthanides (La, Ce, Pr, Nd, Pm, Sm, Eu, Gd, Tb, Dy, Ho, Er, Tm, Yb, and Lu) in addition to Sc and Y [2]. In recent years, metal complexes, including rare earth complexes, have played important roles in popular fields, such as optics, biomedicine, new energy lasers, fibre optic communication, immunoassays, fluorescent lasers, sensors, anticounterfeiting, and organic light-emitting diodes (OLEDs) [3,4,5,6,7,8,9,10,11,12,13]. Neodymium, ytterbium, and erbium complexes can emit near-infrared light and have many valuable applications in night vision and monitoring, medicine, scientific research and industrial detection, optical integrated sensing and communication, and many other fields [14,15,16]. Europium, terbium, and dysprosium complexes can emit light in the visible range. Europium complexes exhibit strong red luminescence. Terbium complexes emit green light, are suitable for use as green light sources in display and lighting devices, and can significantly improve the stability and lifespan of luminescent materials. Dysprosium complexes can emit yellow light. These rare earth complexes with different luminescent properties add rich colour layers to luminescent materials. Since 1942, when Weissman et al. [17] discovered that β-diketone europium complexes emit the characteristic fluorescence of Eu^3+^ ions after absorbing UV excitation light, rare earth-β-diketone complexes have been a focus of research [18,19,20,21,22].

Europium complexes are usually formed by coordination between ligands and metal ions that provide lone-pair electrons to form coordination bonds. The main ligands for europium complexes include β-diketones, organic carboxylic acids, N-heterocyclic ligands, alcohols, and phenols.

β-Diketone ligands, such as acetylacetone and benzoylacetone, are very common, and they are coordinated to Eu^3+^ through two oxygen atoms. Organic carboxylic acid ligands such as benzoic acid and terephthalic acid are coordinated to Eu^3+^ via oxygen. Nitrogen heterocyclic ligands, such as 1,10-phenanthroline (phen) and 2,2′-bipyridine (bpy), coordinate to metal ions via lone-pair electrons on the nitrogen atom. Alcohol and phenol ligands, such as ethanol and phenol, coordinate to Eu^3+^ through oxygen atom coordination.

Organic carboxylic acids and β-diketones, as the most common ligands, have received much attention because they form europium complexes with high kinetic and thermodynamic stability. The ligand structure plays a decisive role in energy transfer from the ligand to the Eu^3+^ centre. The ligand also strongly influences the stability and fluorescence efficiency of europium complexes, so designing and synthesizing ligands that can effectively transfer energy to the Eu^3+^ centre is very important.

Two types of complexes, organic carboxylic acid and β-diketone complexes, have good luminescent properties and stability. Compared to metals in the d-block, Eu(III) has special spectral characteristics [23]. The absorption and emission bands are very narrow, and Eu(III) complexes are designable, easy to prepare and easy to modify, with excellent fluorescence properties, high colour purity, a long fluorescence lifetime, a high quantum yield, and an emission spectral range covering the visible and near-infrared light regions [24,25]. Many studies have focused on the luminescence of rare earth (III) complexes in the visible region and corresponding applications. This paper summarizes the luminescence properties of the red-emitting rare earth europium, the luminescence of β-diketone and organic carboxylic acid–europium complexes and their applications in some fields and describes existing problems and prospects.

## 2. Principle of the Luminescence of Rare Earth Complexes

According to their luminescence mechanism, rare earth complexes can be divided into two main categories: [26] f–f transition luminescent compounds and d–f transition luminescent compounds. For f–f transition luminescent compounds, such as Eu(III) and Tb(III) compounds, luminescence arises from the forbidden f–f transition of the metal ion centre. Compared with non-rare earth compounds, this kind of complex is characterized by little influence of the matrix on the emission wavelength, rich emission spectral lines, a long excited-state life, low-concentration/low-temperature quenching, low luminescence efficiency in the ultraviolet region, and a small absorption coefficient. For d–f transition luminescent complexes, such as Ce(III) and Eu(II) complexes, the luminescence comes from the parity-allowed d–f transition of the ion centre, and the d-orbital energy level is strongly affected by the coordination environment. Thus, these complexes are characterized by a broader emission spectrum, a short excited-state lifetime, and an adjustable luminescence colour. Trivalent europium ion complexes mainly undergo the f–f transition.

**Figure 1:**
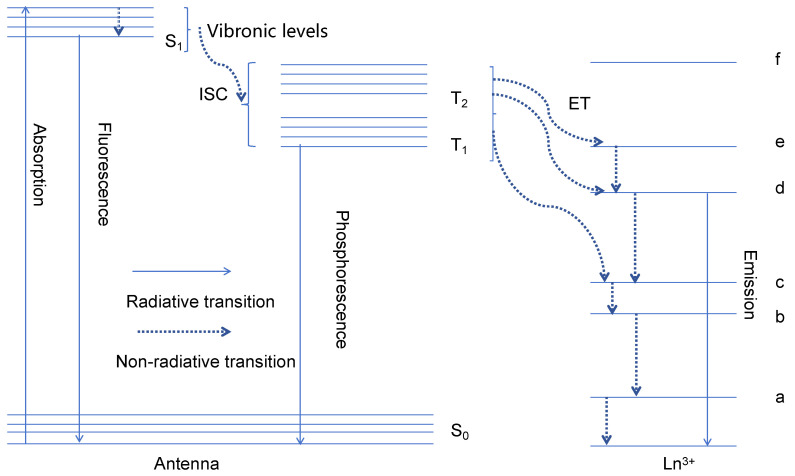
Mechanisms of the antenna effect. S_0_—ground state of the ligand; S_1_—lowest excited singlet state of the ligand; T_1_, T_2_—excited triplet state of the ligand; a–f—rare earth ion energy levels.

The fluorescence of europium(III) complexes is caused mainly by radiationless energy transfer from the ligand and the REE ion. After the central ion receives energy, it emits characteristic fluorescence; this luminescence phenomenon of rare earth ions is called “rare earth-sensitized luminescence”. Because this phenomenon occurs mainly in the central elements of the lanthanide system, such as Sm^3+^, Eu^3+^, Tb^3+^, and Dy^3+^, this phenomenon is also known as “lanthanide-sensitized fluorescence” or “lanthanide-sensitized luminescence”. The ligand-sensitized central ion luminescence effect is called the “antenna effect” [17]. Figure 1 shows the principle of the antenna effect.

Figure 1 shows that for a certain wavelength of excitation light, electrons in the ligand undergo a π → π^*^ transition from the ground state S_0_ to the singlet state S_1_. Following this transition, there can be two energy transfer pathways: (1) S_1_ → S_0_ transition, in which the electrons in the excited state return to the ground state and energy is released in the form of radiation, resulting in molecular fluorescence, and (2) nonradiative transition with crossover from the singlet state to the triplet state. This process can then involve a transition from the triplet state T_1_ back to the ground state S_0_, which is a spin-forbidden transition, resulting in the emission of long-lived phosphorescence (generally at low temperatures). Energy transfer from T1 excites rare earth electrons to higher energy levels, and the electrons undergo a radiative transition back to the ground state, emitting the characteristic fluorescence of the rare earth ion [27,28].

The luminescence of rare earth complexes is also determined by their local crystal field environment. The symmetry ratio (R) quantifies the site symmetry of Eu^3^⁺: R < 1 (e.g., cubic symmetry) favours magnetic dipole transitions (^5^D_0_→^7^F_1_, 590 nm orange emission), while R > 1 (e.g., monoclinic low-symmetry sites) enhances electric dipole transitions (^5^D_0_→^7^F_2_, 613 nm red emission). These mechanisms establish a foundation for the rational design of high-performance rare-earth luminescent materials.

In addition to trivalent europium (Eu^3+^) complexes, luminescent divalent europium (Eu^2+^) complexes also exhibit great potential for use in high-performance OLEDs and other fields due to their unique luminescence mechanism. The luminescence of divalent europium complexes relies primarily on their 5d-4f transition mechanism [29,30,31,32,33]. Compared with traditional f-f transitions, 5d-4f transitions feature allowed transition rules and short lifetimes (on the nanosecond scale), which significantly reduce excited-state quenching, thereby increasing device brightness and mitigating efficiency roll-off. The transition of Eu^2+^ ions from 4f^6^5d^1^ to 4f^7^ is an open-shell electron transition, theoretically allowing for the utilization of 100% exciton energy. The energy of the 5d orbital is influenced by the ligand field, and the emission wavelength can be easily tuned by altering the coordination environment, thereby tuning the luminescent colour. For example, Liu et al. synthesized four macrocyclic Eu^2+^ complexes, EuX_2_-Nn (X = Br, I; n = 4, 8), among which the EuX_2_-N_8_ complex exhibited nearly 100% photoluminescence quantum yield, good air/thermal stability, and grinding-induced colour change properties [34]. Ligand engineering plays a pivotal role in tuning emission properties. For instance, macrocyclic ligands (e.g., 12-crown-4 derivatives) can stabilize Eu^2^⁺ centres, resulting in good photoluminescence quantum yields. In addition, the introduction of fluorinated β-diketones (e.g., F6-acac), which reduce nonradiative decay through their rigid ligand structure, into Eu^2^⁺ complexes can also improve the photoluminescence quantum yield and thermal stability [29,30,31,32,33]. Despite the unique luminescence mechanism and numerous advantages of luminescent divalent europium complexes, stability issues remain a major bottleneck limiting their application. Their stability and performance could be further improved through rational complex design and synthesis strategies, as well as optimization of device structure, thereby promoting their widespread adoption in practical applications. Given the limited information available on this topic, the subsequent sections of this review summarize only the advancements in europium(III) luminescent complexes over the past five years.

## 3. β-Diketone Europium Complexes

**Figure 2:**
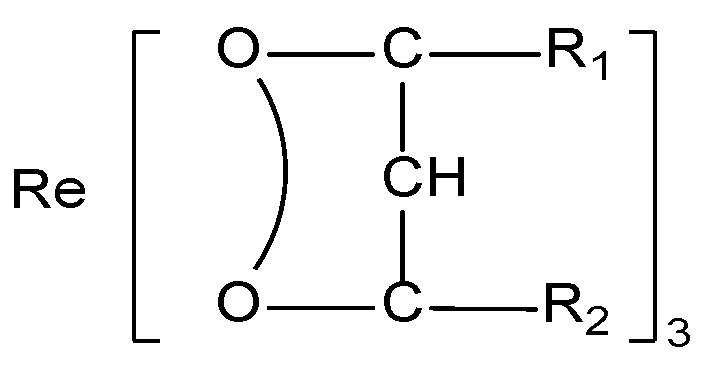
Chemical structure of the β-diketone rare earth complex.

**Figure 3:**
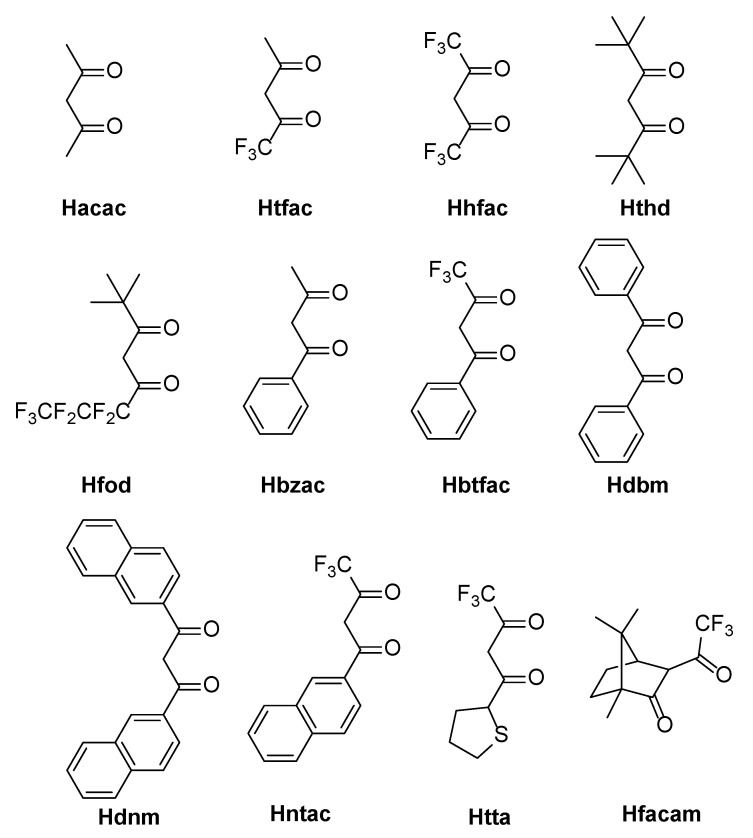
Chemical structure of β-diketones.

Eu^3+^ complexes, especially those containing β-diketones, have been intensively investigated because of their inherent sharp emission peaks and efficient energy transfer. β-diketone and Eu^3+^ complexes have the following structural formula (Figure 2):

Among the components, R_1_ and R_2_ strongly influence the luminescence of the centre ion. When R_1_ is a strong electron-donating group, the luminescence efficiency is significantly improved. The influence follows the order of thiophene > naphthalene > benzene. R_2_ has the strongest sensitization effect when this group is -CF_3_ because the high electronegativity of F can cause the metal–oxygen bond to become an ionic bond. Therefore, some aliphatic hydrocarbon β-diketones containing a -CF_3_ group can cooperate with rare earth ions to produce luminescence. Some common β-diketones are thenoyltrifluoroacetone (TTA), benzoyltrifluoroacetone (BTFA), β-naphthoyltrifluoroacetone (β-NTA), and 1,1,1-trifluoro-5-tert-butyl-2,4-diketone (PTA). Figure 3 shows the common β-diketone ligands.

**Figure 4:**
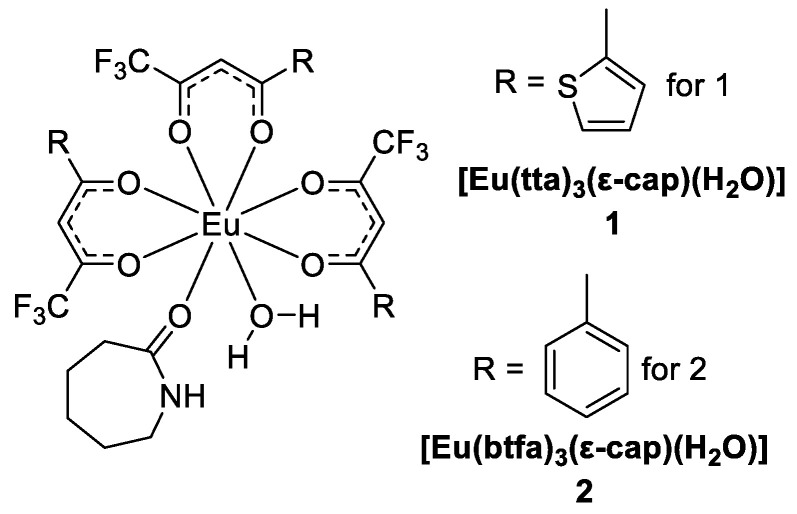
Chemical Structures of **1** and **2**.

Gomes et al. [35] reported two new Eu^3+^ β-diketonate complexes, [Eu(tta)_3_(ε-cap)(H_2_O)] (Complex **1**) and [Eu(btfa)_3_(ε-cap)(H_2_O)] (Complex **2**), which are supported by 4,4,4-trifluoro-1-(2-thienyl)-1,3-butanedione (Htta) or 4,4,4-trifluoro-1-phenyl-1,3-butanedione (Hbtfa) as the primary ligand and ε-caprolactam as the secondary ligand (Figure 4). These ligands act as effective sensitizers of the Eu^3+^ ion, producing intense red radiation. Therefore, Compounds **1** and **2** were selected as markers for 0.38 calibre revolver and 0.380 pistol ammunition. Forensic identification revealed visible luminescent gunshot residue (LGSR). T_1_ → ^5^D_1_ and T_1_ → ^5^D_0_ are the main channels for intramolecular energy transfer in these Eu^3+^ β-diketonate complexes, where compound 1 has a higher quantum yield (41.39%) and quantum efficiency (42.64%). Thus, these Eu^3+^ β-diketonate complexes are effective ammunition markers and could be used to effectively establish the facts of a crime (see Table 1 below).

**Figure 5:**
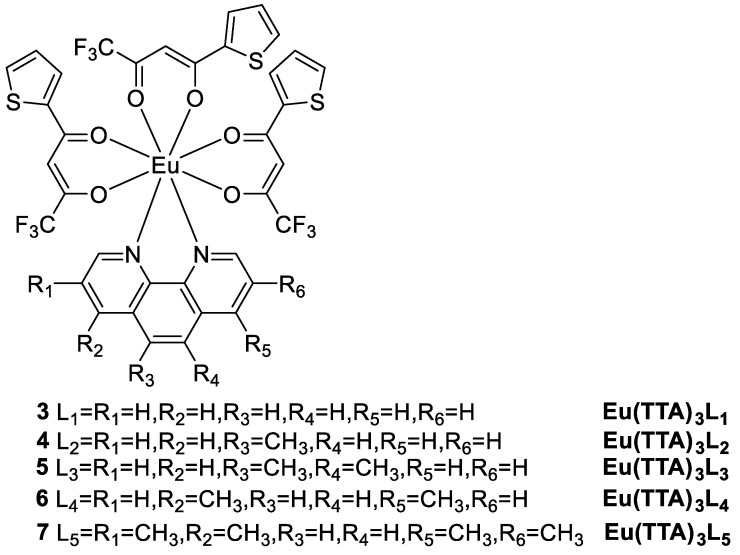
Chemical structures of **3**–**7**.

β-Diketone lanthanide complexes have been extensively studied due to their intense luminescence. However, these complexes are often ligand-unsaturated. Essahili et al. [36] comprehensively assessed the long-term stability of europium β-diketonate complexes. The photoluminescence quantum efficiencies of the five tested complexes are excellent, and the range of quantum yields depends on the medium: from 52% to 55% in DCM solution, from 32% to 72% in the solid state, and from 70% to 85% in a PMMA matrix. These results can be attributed to the presence of chromophores (TTA and phenanthroline ligands) in Eu(TTA)_3_L_1-5_ (Complexes **3**–**7**) (Figure 5), which are directly involved in the sensitization of Eu^3+^ complexes. The use of 2-thenoyltrifluoroacetone (TTA) as the primary ligand and phenanthroline derivatives as secondary ligands resulted in improved quantum yield and stability. In particular, Complex **7** had a high quantum yield of up to 85% at 5% wt PMMA and greater resistance to degradation than the other complexes (see Table 1 below).

**Figure 6:**
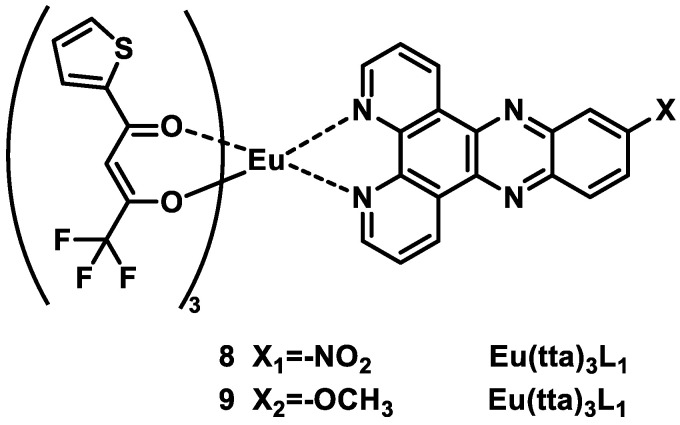
Chemical structures of **8** and **9**.

**Figure 7:**
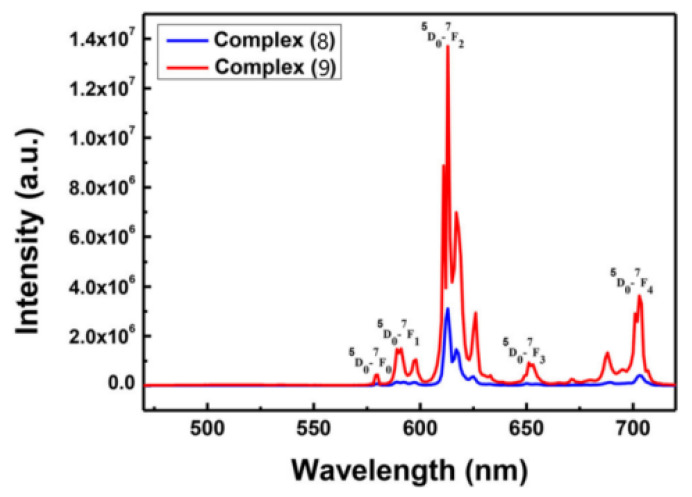
Emission spectra of Complexes **8** (λ_ex_ = 310 nm) and **9** (λ_ex_ = 425 nm) at 298 K in the solid state [37].

Song and coworkers [37] synthesized two europium(III) complexes, Eu(tta)_3_L_1_ (L_1_ = 11-nitrodipyrido [3,2-a:2′,3′-c]phenazine) (Complex **8**) and Eu(tta)_3_L_2_ (L_2_ = 11-methoxydipyrido [3,2-a:2′,3′-c]phenazine) (Complex **9**) (Figure 6), both of which produce red emission corresponding to the ^5^D_0_ → ^7^F_j_ (j = 0–4) transition in different solutions and in the solid state (Figure 7) [37]. Complex 9, with electron-donating groups, has better luminescent properties than Complex 8, with electron-absorbing groups. The quantum yield of Complex **9** (6.7%) is greater than that of Complex **8** (2%) in DMSO, and the fluorescence lifetime of Complex **9** is longer than that of Complex **8** in MeOH, ACN, DCM, and the solid state. Both complexes have strong intracellular luminescence and can be used for fluorescence imaging of living cells (see Table 1 below).

Micro- and nanoplastic pollution has potential ecological and human health risks and is currently an emerging issue of great concern in global environmental science. Luo et al. [38] confirmed that submicron- and even micron-sized plastic particles can be absorbed, accumulated, and transported in crops and vegetables and discovered the channels and mechanisms for the uptake of micro- and nanoplastic particles in plants. Compared with the fluorescence lifetime of nanoseconds for ordinary compounds, rare earth–organic fluorescent compounds have a long fluorescence lifetime (milliseconds), and the use of time-resolved techniques to collect fluorescence signals can significantly reduce background fluorescence interference from biological samples. Luo et al. [39] doped the europium complex Eu(TTA)_3_ (Complex **10**) into the interior of 200 nm polystyrene microspheres (PS-Eu) via the solvation method. The time-resolved fluorescence of the rare earth complexes was utilized to accurately visually track PS-Eu particle uptake and accumulation in plants (wheat and lettuce). In this study, the uptake and translocation of PS-Eu particles in lettuce and wheat were further indirectly quantified and analysed via ICP–MS detection of the Eu content.

**Figure 8:**
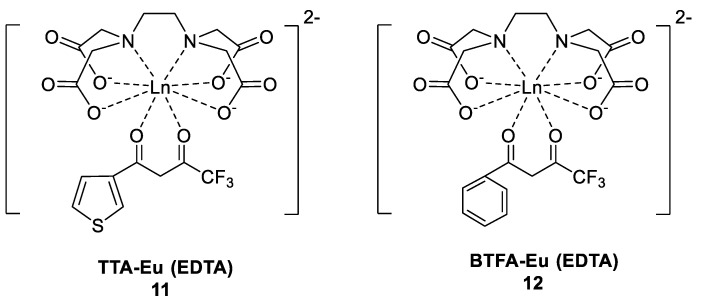
Chemical structures of **11** and **12**.

Silva and others [40] systematically investigated the characteristics of water-soluble β-diketone-Eu-EDTA complexes formed by the addition of ligands to aqueous solutions of Eu-EDTA complexes (Figure 8), where the β-diketones were TTA (Complex **11**) and BTFA (Complex **12**). The Eu-EDTA complex solution exhibited very weak red emission under UV excitation. When a ligand was added to the Eu-EDTA complex in aqueous solution, the water-soluble β-diketone-Eu-EDTA complex exhibited a high luminescence intensity even in dilute conditions, which was attributed to the effective intermolecular energy transfer between the β-diketo acid ligand and lanthanide ions; the results indicated that β-diketone lanthanide complexes could be used as chemical sensor materials in biomedical applications. In addition, the excitation spectrum changes when the molar ratio of the β-diketone ligand to Eu-EDTA is changed; as the molar ratio increases, the excitation maximum gradually redshifts, and the luminescence intensity sharply decreases (see Table 1 below).

**Figure 9:**
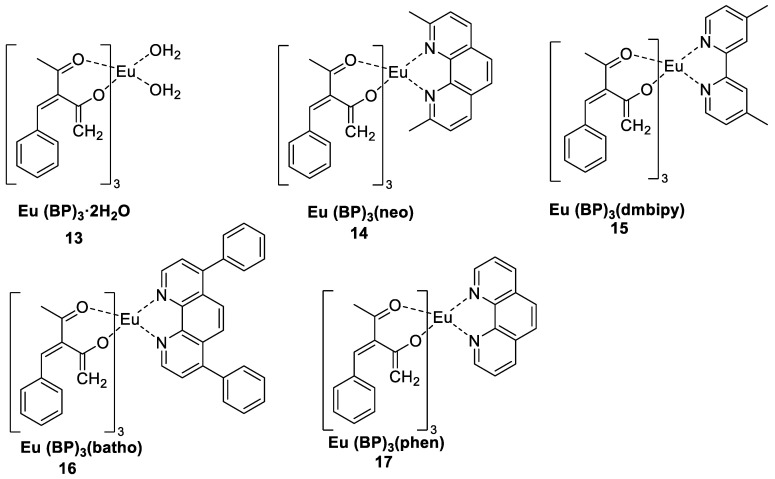
Chemical structures of **13**–**17**.

Doon et al. [41] synthesized binary and ternary red luminescent Eu^3+^ complexes using 3-benzylidene-2,4-pentanedione (BP) as the primary ligand and 1,10-phenanthroline, bathophenanthroline, neocuproine, and 4,4′-′dimethyl-2,2′-′bipyridyl as secondary ligands (Figure 9). The peak at 619 nm corresponding to the ^5^D_0_ → ^7^F_2_ transition is the most intense peak in the emission spectrum. The electric dipole emission transition ^5^D_0_ → ^7^F_2_ is the main reason for the brilliant red light of the Eu^3+^ complexes. The intensity of this ultrasensitive transition is affected by subtle changes in the symmetry of the ligand around Eu^3+^ and the nature of the ligand. The luminescence of the ternary complexes (Complexes **14**–**17**) is much greater than that of the binary complex (Complex **13**), suggesting that the auxiliary ligand protects the Eu^3+^ ion from radiationless decay. The luminescence intensity of Complex **17** is nearly five times greater than that of the other tested luminescent complexes, and the quantum yield reaches 46.29%. The excellent thermal stability of this series of Eu^3+^ complexes makes them promising candidates for OLEDs. The optical bandgaps lie within the range of wide bandgap semiconductors, suggesting that these materials could be used in military radar and biomarker applications (see Table 1 below).

Numerous rare earth complexes exhibit specific emission wavelengths and have unique applications in many fields. The luminescence efficiency of rare earth complexes is influenced by various factors, including the structure of the complex, preparation conditions, and application scenario. According to current research, europium complexes are among the rare earth metal complexes with the highest luminescence efficiency.

**Figure 10:**
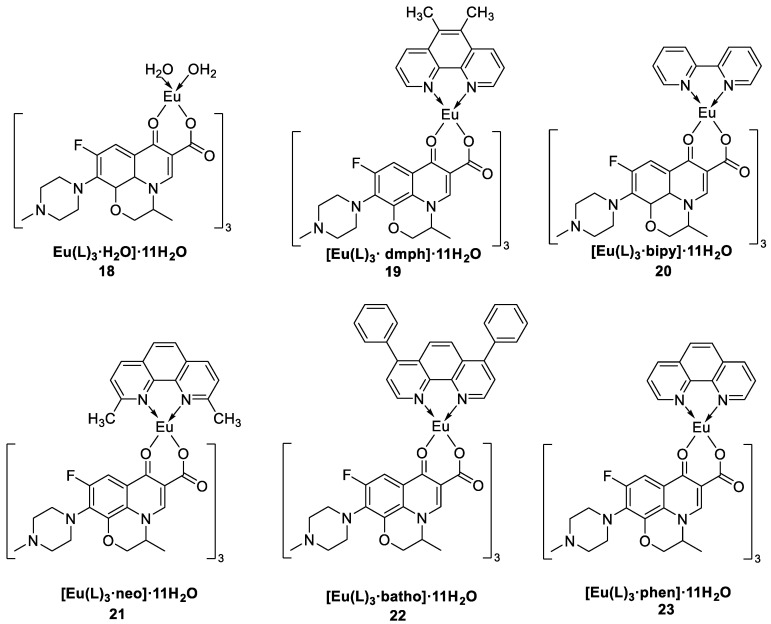
Chemical structures of **18**–**23**.

**Figure 11:**
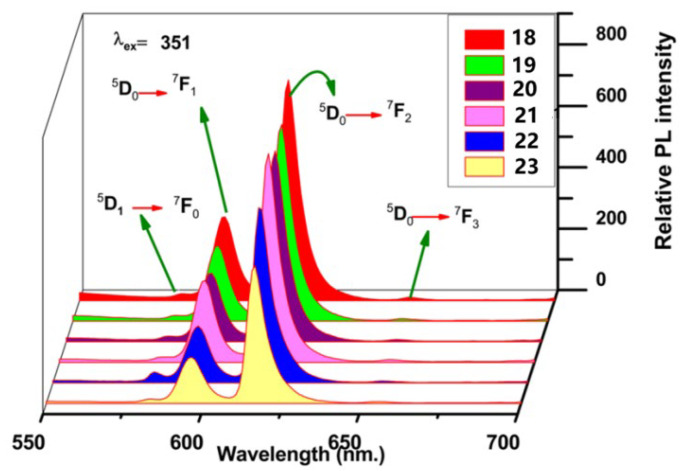
Luminescence emission spectra of Complexes **18**–**23** under excitation at 351 nm [42].

Kumari et al. [42] synthesized a series of complexes (Complexes **18**–**23**) via a solvent-assisted grinding method using β-hydroxyketocarboxylic acid (L) as the primary ligand and 1,10-phenanthroline (phen), 5-6-dimethyl-1,10-phenanthroline (dmph), neocuproine (neo), bathophenanthroline (batho), 2,2′-bipyridyl (bipy), and europium nitrate hexahydrate as secondary ligands (Figure 10). The emission spectra, excitation spectra (Figure 11) [42] and decay time curves were analysed. The secondary ligands tend to bind with the lanthanide metals, which reduces the nonradiative energy loss and thus increases the luminescence intensity. Photoluminescence analysis revealed that the secondary ligand has a synergistic effect and can act as a remarkable sensitizer, effectively transferring energy to the primary ligand. The quantum yield is also significantly elevated, with the highest quantum yield of 54.37% for Complex **23** and the lowest quantum yield of 46.46% for Complex **18**. According to the thermogravimetry–differential thermogravimetry (TG–DTG) curves, the complexes have high thermal stability. The curves show three consecutive decomposition steps in the temperature ranges of 30 → 130 °C, 244 → 419 °C, and 419 → 936 °C. In the temperature range of 130 °C to 244 °C, the TG curve shows negligible decomposition. Therefore, these complexes have excellent stability up to 244 °C. All of these complexes produce warm red emission with high colour purity (the colour purities of all the complexes are in the range of 89.97–97%). and can be applied as red components to produce white light. Owing to their biological properties, these complexes can be used as antibacterial, antifungal and antioxidant agents. The above findings prove that europium complexes can aid in the development of light-emitting devices, white light-emitting diodes, lasers, and solar energy devices (see Table 1 below).

**Figure 12:**
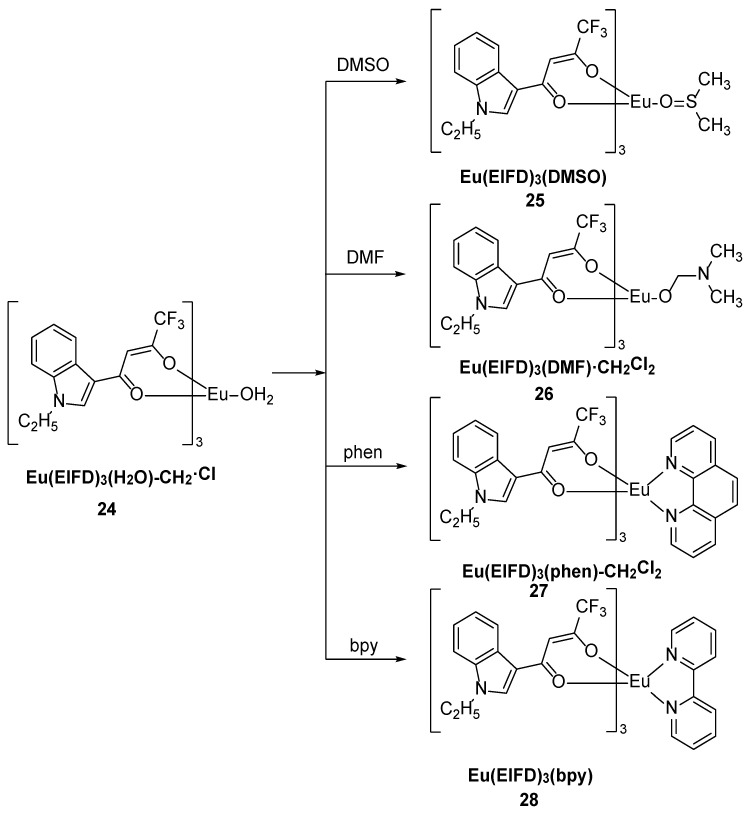
Chemical structures of **24**–**28**.

Li and coworkers [43] synthesized five β-diketone ligand EIFD-Eu^3+^ complexes, namely, Eu(EIFD)_3_(H_2_O)-CH_2_·Cl_2_ (Complex **24**), Eu(EIFD)_3_(DMSO) (Complex **25**), Eu(EIFD)_3_(DMF)·CH_2_Cl_2_ (Complex **26**), Eu(EIFD)_3_(phen)-CH_2_Cl_2_ (Complex **27**), and Eu(EIFD)_3_(bpy) (Complex **28**), where EIFD is 1-(1-ethyl-1H-indol-3-yl)-4,4,4-trifluorobutane-1,3-dione, phen = 1,10-phenanthroline, and bpy = 2,2′-bipyridine (Figure 12). Their photoluminescence properties were also systematically investigated, and the results further indicated that EIFD is an effective sensitizer for the luminescence of the central ion. Complex **24** has the lowest quantum yield (14.47%) due to the presence of an OH oscillator near the Eu^3+^. Luminescence can be effectively suppressed by vibrational relaxation. In contrast, the substitution of solvent molecules with auxiliary ligands containing nitrogen and oxygen in Complexes **25**–**28** increases the quantum yield; among these compounds, the highest luminescence quantum yield of 32.54% is observed for Complex **28** (see Table 1 below).

**Figure 13:**
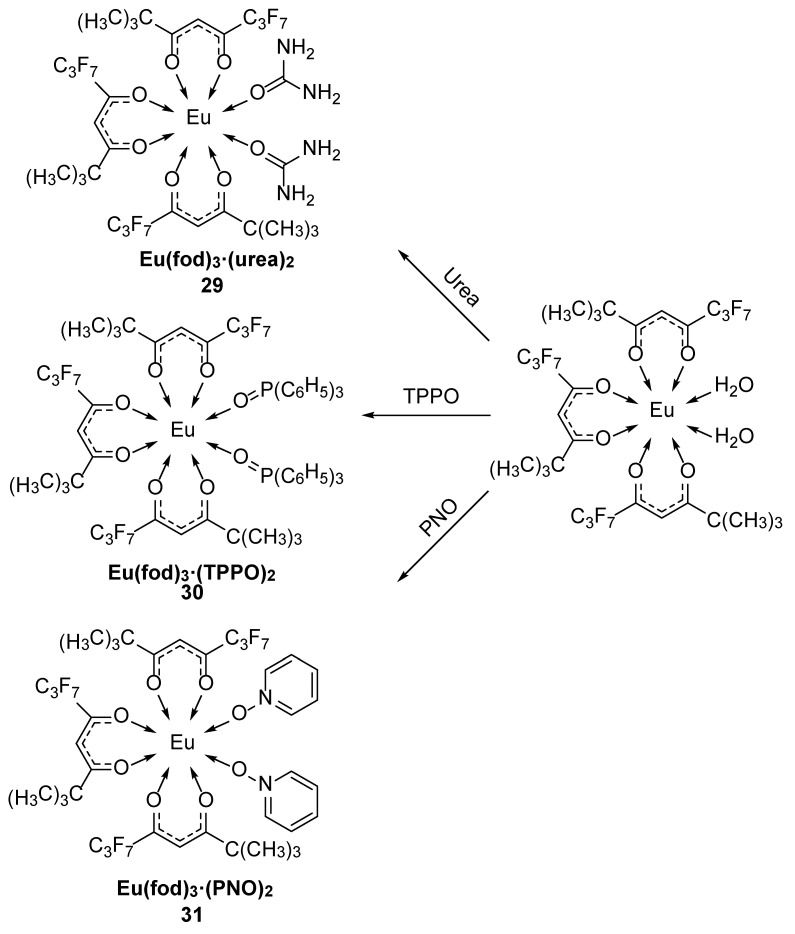
Chemical structures of **29**–**31**.

Singh et al. [44] used 2,2-dimethyl-6,6,7,7,8,8,8-heptafluororo-3,5-octanedione (fod)-fluorinated β-diketone as the primary ligand and urea, triphenylphosphine oxide (TPPO) and pyridine-N-oxide (PNO) as secondary ligands to synthesize ternary complexes (Complexes **29**–**31**) (Figure 13). Emission measurements revealed that the hypersensitive electric dipole ^5^D_0_ → ^7^F_2_ transition of Eu^3+^ was characterized by strong emission in the red spectral region. To determine the role of the auxiliary ligands, the characteristics of the increase in luminescence intensity were comprehensively compared. The auxiliary ligand not only replaces the solvent molecule in the coordination sphere but also indirectly sensitizes the metal ion by transferring energy from the triplet state of the ligand to the emission energy level of the corresponding Ln^3+^ ion, thus increasing the luminescence intensity. The prepared metal complexes form a cage-like structure around the central metal ion, which effectively absorb energy and transfers it to bonded metal ions via the bulky fluorinated chelating ligands. Eu^3+^ sensitization by the auxiliary ligands is in the order of urea < TPPO < PNO. The authors also used a fluorinated β-diketone (4,4,5,5,6,6,6-heptafluoro-1-(2-thienyl)-1,3-hexanedione) (Hfth) as the primary ligand [45] and TPPO, PNO and 2-pyridinium-N-oxide as secondary ligands to prepare red luminescent materials (Complexes **32**–**34**). Luminescence analysis of the synthesized complexes revealed that the increase in the luminescence intensity was due mainly to the introduction of asymmetric auxiliary ligands around the central lanthanide ion and to increased stability between Ln^3+^ and the auxiliary ligands. In both experiments, the complexes containing PNO had the highest quantum yields, 49% and 52%, and the longest fluorescence lifetimes, 0.840 ms and 0.43 ms, respectively. Auxiliary ligands such as urea have high-energy O–H and N–H vibrations, which result in excellent luminescence properties of the resulting materials for OLED applications. In the second experiment, the interactions between the metal complexes resulted in significant antioxidant effects (see Table 1 below).

**Figure 14:**
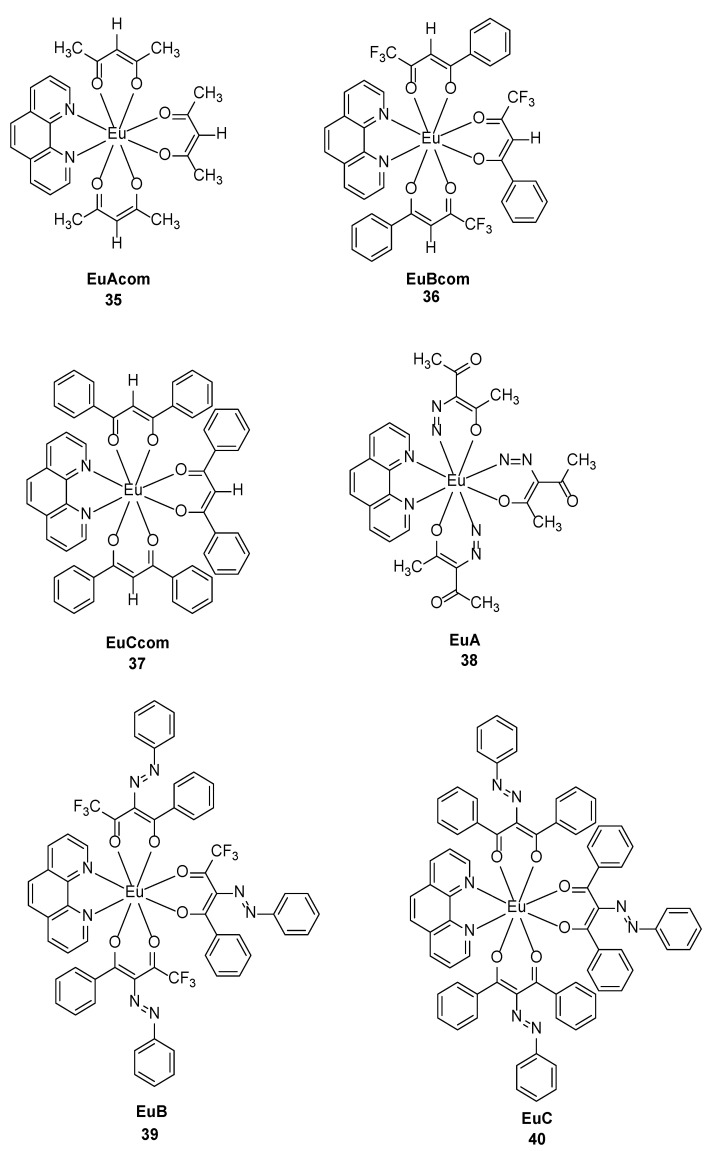
Chemical structures of **35**–**40**.

Lily Arrué et al. [46] synthesized six complexes with β-diketone derivatives as ligands (Complexes **35**–**40**) and Eu(Phen)(X)_3_ (X = β-diketone) (Figure 14) and demonstrated that some antenna ligands (Bcom and Ccom) can effectively bind to the europium centre. The emission properties can be altered by modifying the structural features of the antenna ligands. The hypersensitive transition, which is an electric dipole-induced transition, is sensitive to the coordination environment of Eu^3+^. The most intense transition corresponds to ^5^D_0_ → ^7^F_2_, causing the complex to emit bright red light. When an azo group is present in EuA, the luminescence of the corresponding complex is weakened, and EuAcom produces a stronger luminescence band at approximately 600 nm. The incorporation of azo groups into β-diketones decreases the quantum yield of the europium emission band (where the quantum yield of the EuA complex with incorporated azo groups is 0.007% and that of EuAcom is 0.01%) owing to structural changes resulting in a shift in the triplet state, such as the identified sensitization pathway. Therefore, ligands that are asymmetric, have electron-donating groups, have no quenching bonds, and meet energy requirements are the best choice for achieving high europium luminescence efficiency (see Table 1 below).

**Figure 15:**
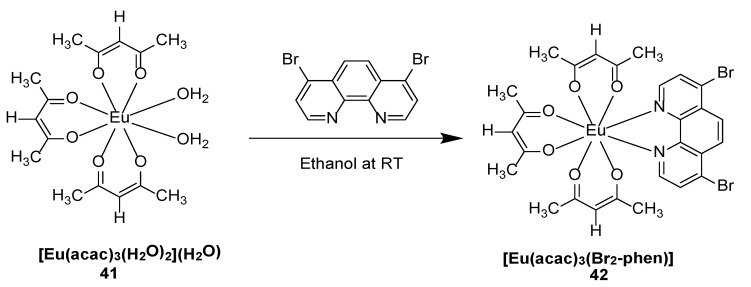
Chemical structures of **41** and **42**.

Ilmi and coworkers [47] synthesized two novel octa-coordinated red-emitting complexes [Eu(acac)_3_(H_2_O)_2_](H_2_O)) and ([Eu(acac)_3_(Br_2_-phen)] [acac: acetylacetonate, Br_2_-phen: 4,7-dibromo-1,10-phenanthroline] (Complexes **41**–**42**) (Figure 15). The intramolecular energy transfer (W_ET_) and inverse energy transfer (W_BT_) follow the pathway S_0_ → S_1_ → T_1_ → ^5^D_1_ → ^5^D_0_ → ^7^F_0.4_. The third transition in the series (^5^D_0_ → ^7^F_2_, 16,231 cm^−1^) is hypersensitive; therefore, its strength is more affected by subtle variations in the local symmetry of Eu^3+^ and the nature of the ligand. The emission spectrum of [Eu(acac)_3_(Br_2_-phen)] is dominated by the hypersensitive ^5^D_0_ → ^7^F_2_ transition in both the solid state and in DCM solution. This complex has a large excited emission cross section, which is promising for high-power laser applications (see Table 1 below).

**Figure 16:**
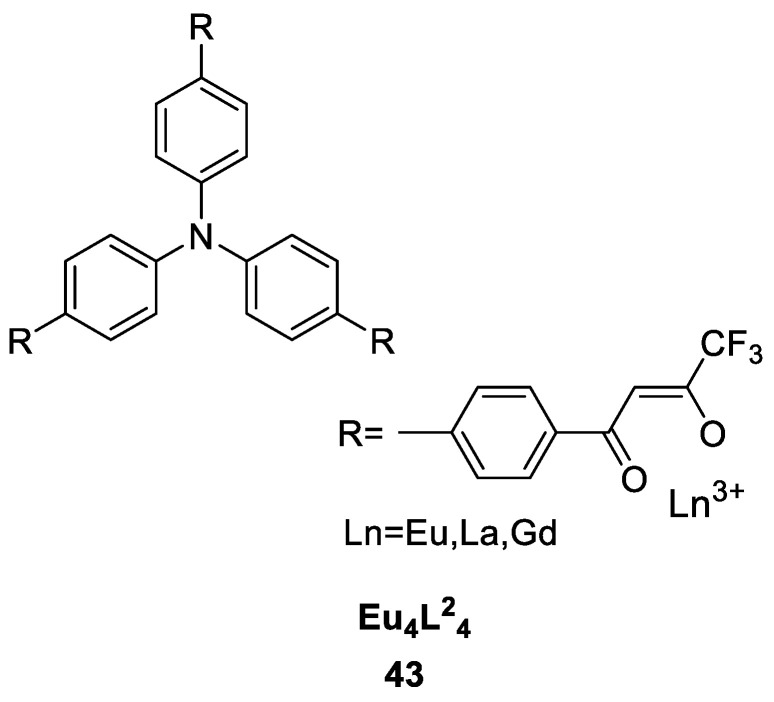
Chemical structure of **43**.

Biogenic amines (BAs), such as ethylenediamine, putrescine, cadaverine, and spermine, are usually produced in spoiled food and are very hazardous to human health. Li et al. [48] utilized a Eu(III) tetrahedral cage structure with 3-β-diketone (Eu_4_L^2^_4_) as a luminescent sensor for volatile BAs (Figure 16) (Complex **43**). The complex exhibited two well-resolved absorption bands in the UV/visible absorption spectral range of 280–450 nm. The observed maximum emission of Eu^3+^ ions at 613 nm revealed that when used as an antenna, L^2^ can effectively sensitize Eu^3+^ ion luminescence. Under 390 nm excitation, five characteristic emission bands of the Eu^3+^ ion were observed at 578, 592, 613, 650 and 702 nm, corresponding to the ^5^D_0_ → ^7^F_J_ (J = 0–4) transition. The luminescence quantum yield of the Eu(III) centre was measured to be 3.55%. The luminescence response of BAs is attributed to the nucleophilic interaction of the amine group with the trifluoroacetyl carbon atom in the ligand. The large cavity and rhombic opening at the edges of the tetrahedron ensure good permeability of the film to BAs, which is responsible for the fast response time. The experimental results confirm the utility of this cage structure complex as a sensing material for food spoilage monitoring (see Table 1 below).

**Figure 17:**
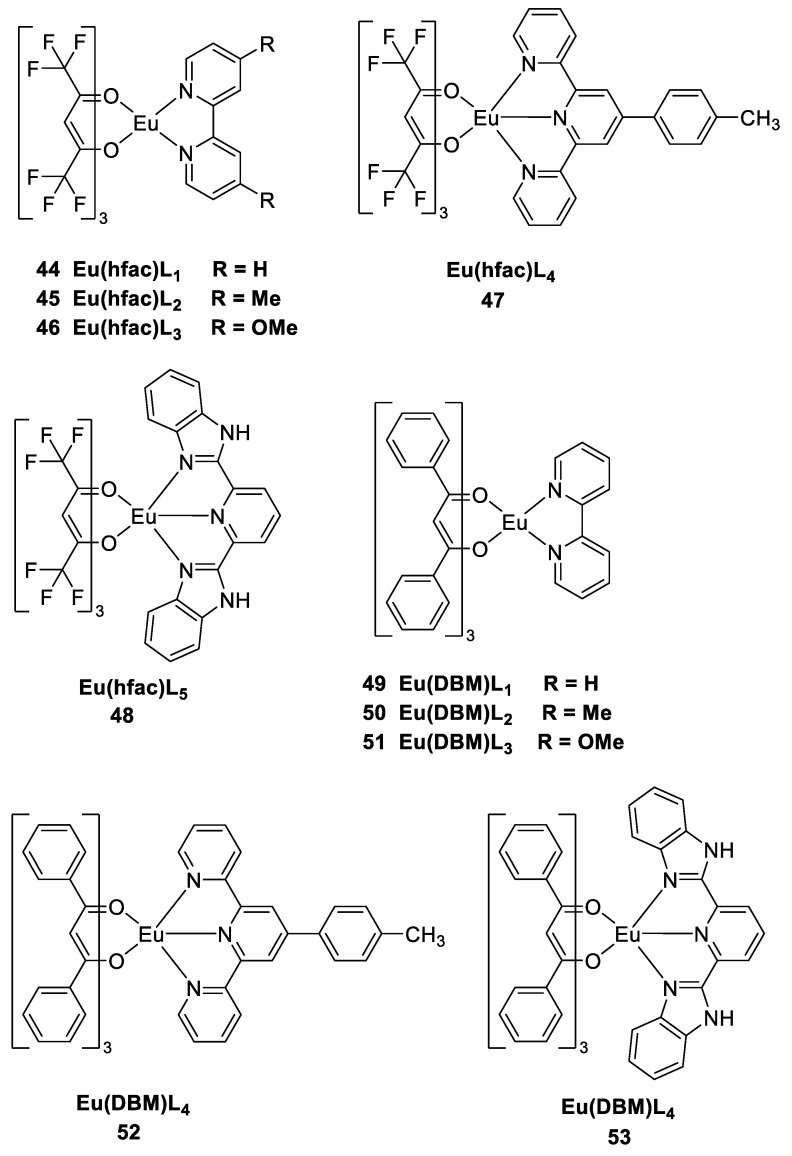
Chemical structures of **44** and **53**.

Scientists are making continuous efforts to utilize renewable energy sources to reduce the human impact of climate change and address the growing global energy demand. Luminescent solar concentrators (LSCs) based on europium β-diketonate have attracted great attention as a promising solar energy harvesting technology. These LSCs utilize specially designed europium complexes with β-diketonate ligands that exhibit remarkable luminescence properties to enhance the absorption and concentration of sunlight [49]. Essahili et al. [50] selected two β-diketo acid ligands, dibenzoylmethane (DBM) and hexafluoroacetone (hfac), to synthesize a series of europium β-diketo acid complexes based on bipyridine and tripyridine derivatives to study the long-term photoluminescence stability of PMMA-doped films. The complexes exhibit a distinct red spectral emission peak at 613 nm and a high photoluminescence quantum yield. The complexes were doped into PMMA films, and higher thermal resistance was observed for Eu(hfac)L_1-5_ (Complexes **44**–**48**) than Eu(DBM)L_1-5_ (Complexes **49**–**53**) (Figure 17). To assess their stability, the films were stored under light, room, and dark conditions for 720 h, and the photoluminescence was recorded at 0 h and 720 h. Compared with DBM-based europium complexes Eu(DBM)_3_L_1-5_, the hfac-based europium complexes Eu(hfac)_3_L_1-5_ exhibited good stability under light, room and dark conditions. The hfac-based complexes show excellent performance in terms of fluorescence lifetime, and the developed hybrid materials have great potential for applications in high-efficiency solar energy conversion and lay the foundation for further developments in this field (see Table 1 below).

**Figure 18:**
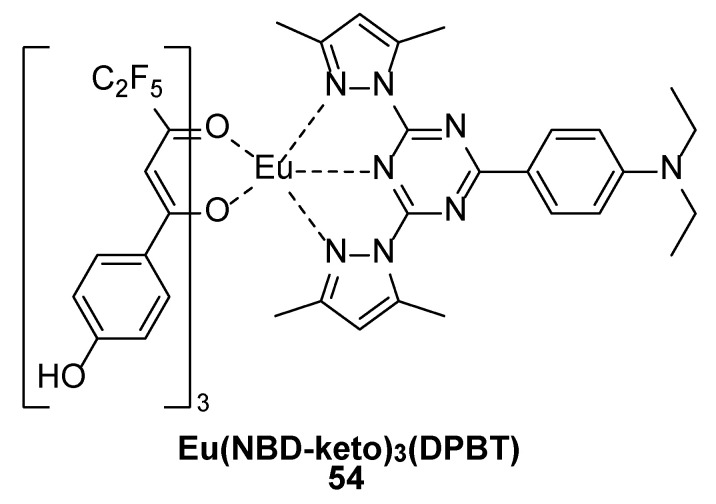
Chemical structure of **54**.

The presence of small molecules such as cysteine (Cys), homocysteine (Hcy), and glutathione (GSH) in the body is indicative of certain human diseases. Song et al. [21], considering the excellent luminescence properties of Eu^3+^ complexes, designed and synthesized an NBD-conjugated β-diketonate-Eu^3+^ complex [Eu(NBD-keto)_3_(DPBT)] (Complex **54**) (Figure 18) (NBD-keto: 7-nitro-2,1,3-benzoxadiazole (NBD) conjugated to 1,1,1,2,2-pentafluoro-5-phenyl-3,5-pentanedionate through an “O” ether bond; DPBT: 2-(N,N-diethylanilin-4-yl)-4,6-bis(3,5-dimethylpyrazol-1-yl)-1,3,5-triazine), which is a unique fluorescent probe for the detection and differentiation of biothiols. This Eu^3+^ complex probe has two absorption peaks at 330 nm and 390 nm, and upon reaction with biothiols, the absorption peak at 390 nm redshifts to 400 nm due to the formation of Eu(keto)_3_(DPBT). Eu(NBD-keto)_3_(DPBT), which is not luminescent by itself, reacts with biothiols via intramolecular interactions between NBD and the β-diketo acid–Eu^3+^ group that form the β-diketo acid–Eu^3+^ complex [Eu(keto)_3_(DPBT)], which emits red light at 610 nm. Moreover, green-fluorescent (short-lived) NBD-nR (R = Cys or Hcy) and nonluminescent NBD-sR (R = GSH) emit light at 540 nm. These luminescent response behaviours result in a combination of time-gated and steady-state luminescence modes that can be used for the detection of total biothiols and to differentiate between GSH and Cys/Hcy. GSH and Cys/Hcy in cell lysates were quantitatively detected and identified via this probe, revealing the potential of this probe for biomedical applications (see Table 1 below).

**Figure 19:**
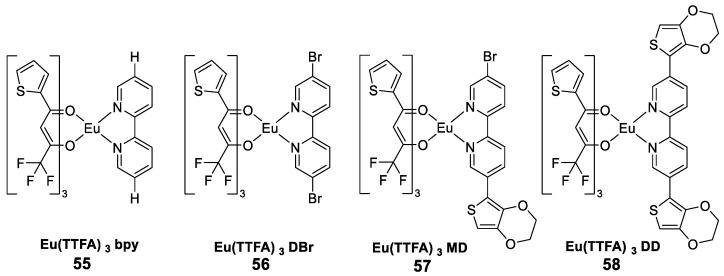
Chemical structures of **55**–**58**.

Dalal et al. [51] synthesized solid ternary europium complexes consisting of fluorinated β-diketones (thenoyltrifluoroacetone, TTFA) and heteroaromatic bidentate auxiliary ligands (Figure 19). The luminescence characteristics of the complex were measured, and it was demonstrated that the ligand effectively sensitized the Eu^3+^ ion through the antenna effect. The photoluminescence excitation spectrum shows that the Eu^3+^ complex is effectively excited in the UV region, and the emission spectrum includes the characteristic peaks of the ^5^D_0_ → ^7^F_2_ transitions of the europium ion, with the strongest emission peak at 611 nm (^5^D_0_ → ^7^F_2_), which is due to the hypersensitive electropositive dipole transition. The emission spectra of Complexes **55**, **56**, **57**, and **58** were obtained at 274, 277, 275, and 277 nm, respectively. The spectral peaks at 579, 591, 611, 653, and 702 nm belong to the ^5^D_0_ to ^7^F_J_ transitions (where J = 0, 1, 2, 3, 4). The overlap of the absorption spectrum of TTFA, the absorption spectrum of the neutral ligand and the excitation spectrum of the europium complex at an emission wavelength of 611 nm confirms the selective and effective sensitization of the Eu^3+^ ion by TTFA in Complex **54** (see Table 1 below).

**Figure 20:**
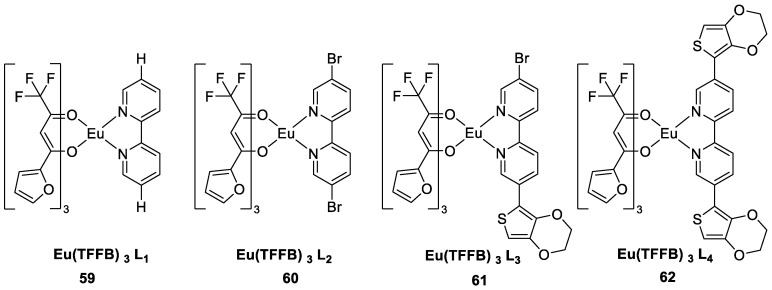
Chemical structures of **59**–**62**.

Singh et al. [52] prepared a series of luminescent Eu(TFFB)_3_X [X = auxiliary component] complexes using the fluorinated β-diketone ligand 4,4,4-trifluoro-1-(2-furyl)-1,3-butanedione (TFFB) as the main sensitizer (Figure 20), and the complexes exhibited luminescence in the visible region under UV excitation owing to the sharp peak of the Eu^3+^ transition. IR and NMR spectroscopic data confirmed the effective binding of the ligand to Eu^3+^. The emission peak of the Eu^3+^ complex at 612 nm (^5^D_0_ → ^7^F_2_) is in the red region. The spectra of the complexes include two absorption bands with different relative intensities at 285 nm and 352 nm because all the complexes contain the same TFFB ligand. The auxiliary ligand plays a minor role here, and the absorption patterns of Complexes **59**–**62** are very similar. The red luminescence of the complexes indicates effective sensitization of Eu^3+^ ions via the antenna effect; furthermore, these materials can be used for the preparation of OLEDs (see Table 1 below).

The luminescence properties of β-diketone europium complexes are related not only to the type of β-diketone ligand but also sometimes to the solvent. In this work, we found that, in general, β-diketone europium complexes containing conjugated aromatic rings have stronger luminescence properties than do aliphatic β-diketone europium complexes. The introduction of hydrophobic groups into the β-diketone ligand reduces the fluorescence burst. When neutral ligands are added as auxiliary ligands (e.g., 2,2′-bipyridine, 1,10-phenanthroline), the neutral ligands can sometimes increase the luminescence intensity. In complexes with the same basic structure, the quantum yield of dinuclear complexes is generally greater than that of mononuclear complexes.

## 4. Europium Complexes with Organic Carboxylic Acids

Owing to their excellent photoluminescence properties and good thermal stability, rare earth–carboxylic acid complexes have attracted much attention. Therefore, these rare earth complexes have a wide range of applications in OLEDs, lasers, fluorescence immunoassays, and other fields. Although the luminescence brightness of rare earth complexes with organic carboxylic acids in OLED materials is not yet as good as that of rare earth complexes with β-diketones, their photostability is better than that of the latter, and their brightness could be improved on the basis of future experiments.

**Figure 21:**
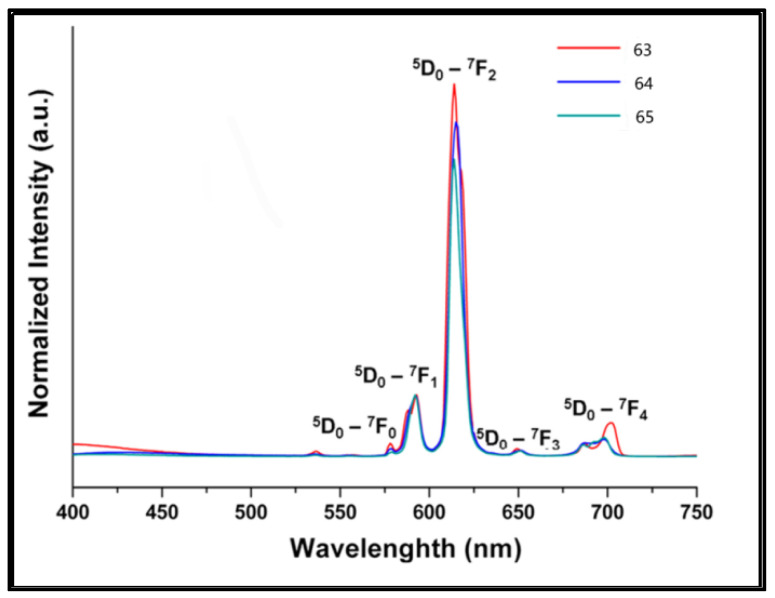
Emission spectra of Complexes **63**–**65** (1–3) excited at 350 nm [53].

Gai et al. [53] prepared {[Eu(ptptc)_0.75_(H_2_O)_2_]-0.5DMF-1.5H_2_O}n (Complex **63**) {[Me_2_H_2_N]_2_[Eu_2_(ptc)_2_(H_2_O)(DMF)-1.5DMF-7H_2_O}_n_ (Complex **64**) and {[Eu(Hptptc)(H_2_O)_4_]-0.5DMF-H_2_O}_n_ (Complex **65**) by reacting europium sulfate octahydrate with *p*-terphenyl-3,3″,5,5″-tetracarboxylic acid (H_4_ptptc) in a mixed solvent system. All of the complexes display bright red luminescence because of the characteristic ^5^D_0_−^7^F_J_ (J = 0–4) transitions (Figure 21) [53]. Complex **63** has a higher quantum yield (22%), which may be related to the dodecahedral structure of Complex **63**, in which the hypersensitive transition ^5^D_0_ → ^7^F_2_ occurs more frequently. Since there are four bound water molecules in Complex **65**, the nonradiative rate constant is 60% larger than that of **64**, leading to a decrease in the total quantum yield (11%). Despite the vibrations of O-H in water molecules, which enhance the nonradiative decay process and decrease the luminescence intensity, Eu^3+^ can still be highly sensitized by the π-rich ligand H_4_ptptc, which acts as a strongly absorbing sensitizer. According to the experimental results, the triplet energy level of the ligand is slightly greater than the emission energy level of Eu^3+^, which indicates that H_4_ptptc is an efficient europium-based red luminescence sensitizer (see Table 1 below).

**Figure 22:**
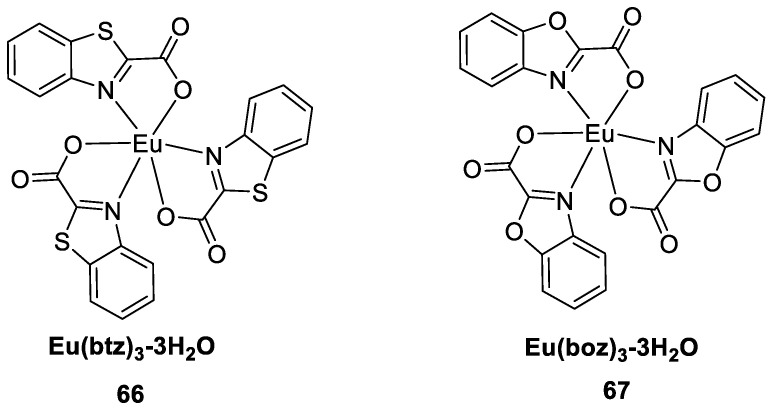
Chemical structures of **66** and **67**.

Koshelev et al. [54] proposed a method for the directed synthesis of novel lanthanide luminescent complexes for use as OLED emissive layer materials by increasing the conjugation length and introducing heteroatoms at appropriate positions in combination with neutral ligands. The introduction of heteroatoms (not only N but also S and O) increases electron mobility, as well as the triplet state energy. Both the bright luminescence of europium ions and electron mobility are ensured. In this study, conjugated heteroaromatic carboxylic acid ligands were used with benzoxazole-2-carboxylate (boz) and benzothiazole-2-carboxylate (btz) as secondary ligands to obtain the complexes Eu(btz)_3_-3H_2_O (Complex **66**) and Eu(boz)_3_-3H_2_O (Complex **67**) (Figure 22). The introduction of the neutral ligands o-phenanthroline (Phen) and bathophenanthroline (BPhen) resulted in a moderate increase (ligand = btz) or a decrease (ligand = boz) in the quantum yield. The highest electroluminescence efficiency was obtained for the complex with the ligand combination btz and Phen (see Table 1 below).

**Figure 23:**
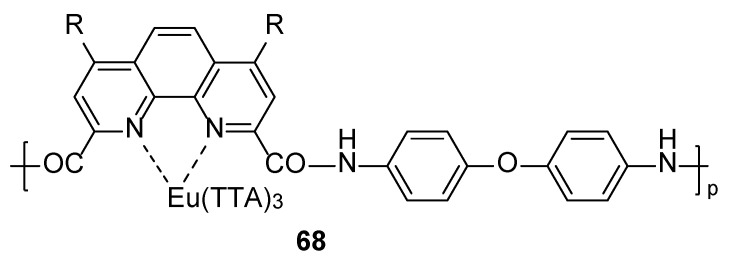
Chemical structure of **68**.

Goikhman and coworkers [55] used dichloroanhydride 1,10-phenanthroline-4,7-dichloro-2,9-dicarboxylic acid as a monomer to obtain strongly thermally stable copolymers (Complex **68**) (Figure 23). The complex with Eu(TTA)_3_ has significant photoluminescence properties in the visible range at 620 nm. The photoluminescence intensity of this copolymer is two times greater than that of similar systems without substituents at the 4th and 7th positions of the phenanthroline fragment, and it has good mechanical properties and thermal stability (see Table 1 below).

**Figure 24:**
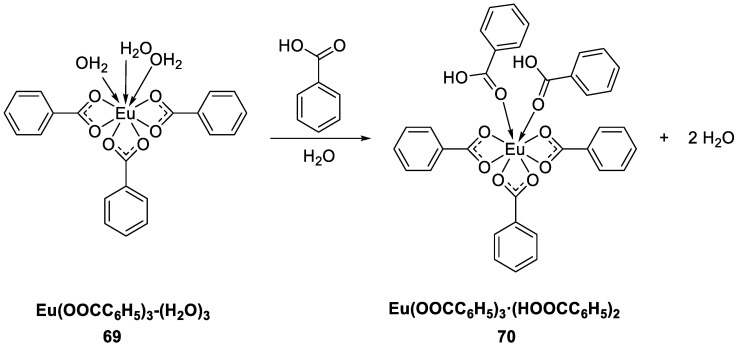
Chemical structures of **69** and **70**.

Hernández-Fuentes et al. [56] synthesized a europium(III) intermediate compound [Eu(OOCC_6_H_5_)_3_-(H_2_O)_3_] (Complex **69**) and a final compound [Eu(OOCC_6_H_5_)_3_·(HOOCC_6_H_5_)_2_] (Complex **70**) using benzoic acid as a ligand (Figure 24). Both compounds show absorption at 290 nm, belonging to the S_0_–S_1_ (π→π^*^) transition, and characteristic red emission at 616 nm (corresponding to the ^5^D_0_ → ^7^F_2_ transition). Owing to the chelation of the two additional ligands, the local symmetry of the Eu^3+^ ion has a greater effect on the hypersensitive ^5^D_0_ → ^7^F_2_ transition band and its intensity in Complex **70** than in Complex **69.** The fully coordinated compound shows a 15% increase in luminescence, indicating that energy loss through the nonradiative pathway is prevented. The fluorescence lifetime increases from 0.42 ms in Complex **69** to 0.53 ms in Complex **70** because the two carboxylic acid ligands act as chelating agents. To some extent, chelation ensures greater transfer of ligand energy to the central ion, preventing energy loss due to nonradiative transitions and thus increasing the decay time (see Table 1 below).

**Figure 25:**
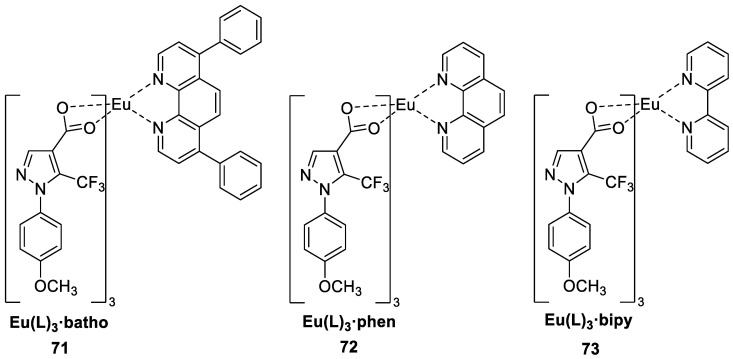
Chemical structures of **71**–**73**.

Khanagwal and others [57] synthesized three red photoluminescent europium complexes (Complexes **71**–**73**) via a solution precipitation method with the fluorinated carboxylate ligand 1-(4-methoxyphenyl)-5-(trifluoromethyl)-1H–pyrazole-4-carboxylic acid (L) as the main ligand and bathophenanthroline, 1,10-phenanthroline and 2,2-bipyridyl as auxiliary ligands (Figure 25). The UV absorption spectra of the complexes are redshifted compared with those of the ligands. The auxiliary ligands not only reduce the burst generated by water molecules but also enhance the effectiveness of energy transfer of some of these complexes, and the quantum yield of the complexes is improved. When water is used as the auxiliary ligand, the quantum yield is only 7.84%, whereas when 2,2-bipyridine is used as the auxiliary ligand, the quantum yield reaches 36.69%, and the fluorescence lifetime significantly improves. In addition, this complex has good antimicrobial and antioxidant properties. Experiments have shown that these complexes have promise in the fields of light-emitting devices, white light diode manufacturing, lasers, and solar energy (see Table 1 below).

**Figure 26:**
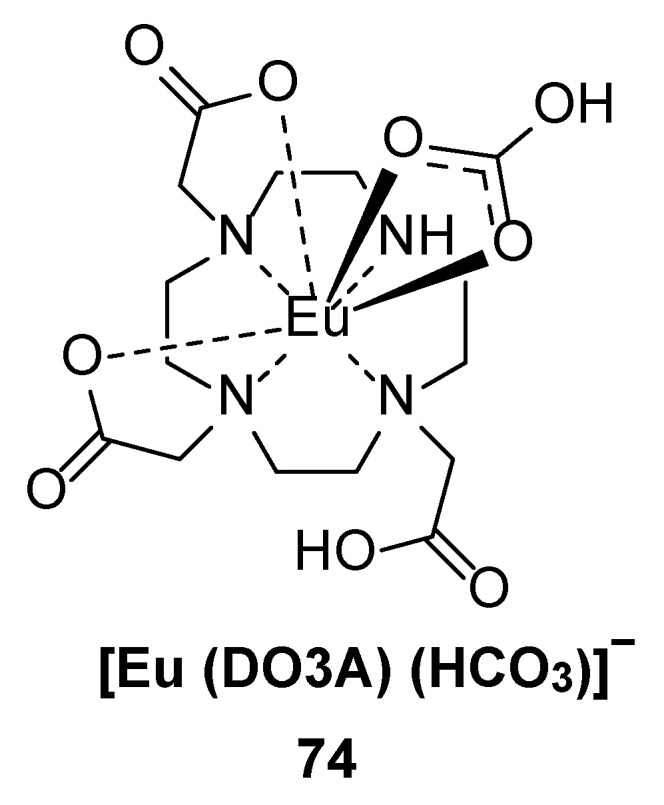
Chemical structure of **74**.

Sorensen et al. [58] studied the luminescence response of the 1,4,7,10-tetraazacyclododecane-1,4,7-triacetic acid (DO3A) Eu^3+^ complex (Complex **74**) (Figure 26) to bicarbonate concentration. The fluorescence lifetime of Eu-DO3A is 0.64 ms in H_2_O and 2.3 ms in D_2_O. With increasing bicarbonate concentration, the emission spectrum of Eu-DO3A changes. The shape and intensity of the ^5^D_0_ → ^7^F_2_ emission bands change, and the luminescence spectrum clearly changes with the bicarbonate concentration. DO3A was evaluated as a luminescent sensor for bicarbonate, and the bicarbonate concentration could be determined when the pH and conductivity of the sample were known (see Table 1 below).

Li et al. [59] prepared a novel Eu^3+^ complex: a dual-ligand Eu^3+^ complex (Complex **75**) of terephthalic acid (TPA) and methylimidazole (MIM). The maximum fluorescence intensity was reached when the molar ratio of TPA to MIM was 1:4. The emission intensity of the complex was the strongest when the excitation wavelength was 280 nm. The complex has unique luminescence properties, and red fluorescence can be observed under 254 nm UV irradiation. The complex also has good stability and dispersibility, and a selective burst in fluorescence emission is induced by acetaldehyde due to the special structure of the complex. After the addition of acetaldehyde, the UV absorption peak of the complex at 240 nm is almost unchanged. However, the UV absorption peak at 283 nm increases in intensity and redshifts to 285 nm, indicating that the addition of acetaldehyde may affect the chemical structure of the complex. On this basis, a simple, effective, low-cost and easy-to-operate chemical sensing method for the detection of trace or ultratrace amounts of acetaldehyde was developed. This method has a good detection limit, linearity, accuracy and precision compared with existing methods and has been applied to the quantitative detection of acetaldehyde in alcoholic beverages and environmental and biological samples, demonstrating promise for future applications (see Table 1 below).

**Figure 27:**
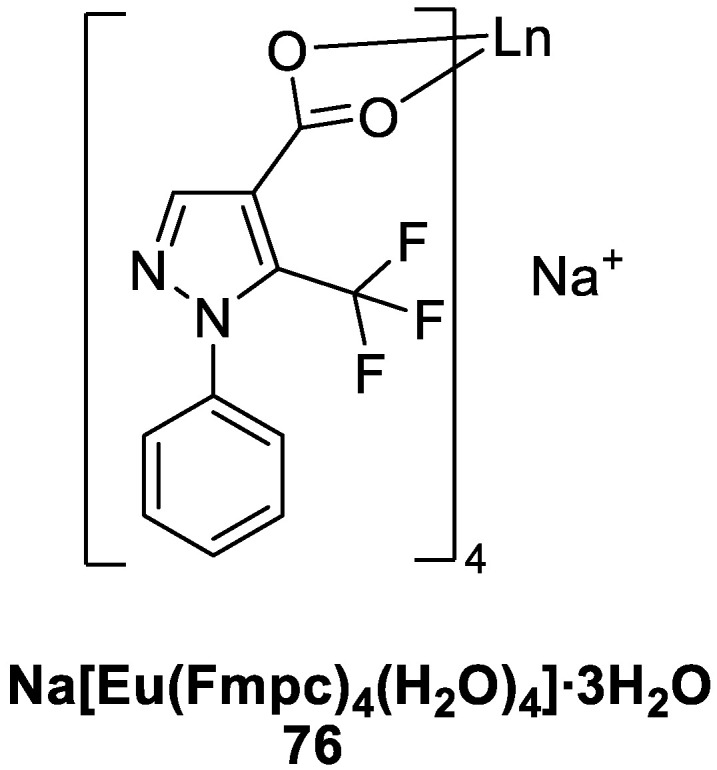
Chemical structure of **76**.

Canisares [60] synthesized a novel, nontoxic, red luminescent complex with carboxylic acid ligands that has high sensitization efficiency: Na[Eu(Fmpc)_4_(H_2_O)_4_]∙3H_2_O (Fmpc: 1-phenyl-5-(trifluoromethyl)-1H-pyrazole-4-carboxylic acid) (Complex **76**) (Figure 27). Experimental studies have shown high sensitization of Eu^3+^ ions by Fmpc ligands, with a sensitization efficiency of 98%. (Na[Eu(Fmpc)_4_(H_2_O)_4_]∙3H_2_O) has good luminescence performance in acetonitrile, and its fluorescence lifetime is 0.76 ms. The relative quantum yield of the complex is 27.9%, and the fluorescence lifetime in aqueous solution is 0.293 ms, with a quantum yield of 4.5%. The Fmpc ligand can effectively transfer the absorbed energy to the Eu^3+^ emission energy levels to produce good luminescence; moreover, it is nontoxic and can be used as a luminescent dye in biological products (see Table 1 below).

Júnior et al. [61] presented the synthesis of a new LnMOF [Eu(Hbtec)]_n_ complex (Complex **77**) (where Hbtec^3-^ is a tetracarboxylate anion from 1,2,4,5-benzenotetracarboxylic acid (H4btec)); the Hbtec^3–^ ligand interacts with the Eu^3+^ centre via three different coordination modes. The energy transfer process of this compound, which has intense red–orange emission and high thermal stability, was determined from the excitation spectrum based on the hypersensitive transition ^5^D_0_ → ^7^F_2_ (λ = 614 nm) at 77 K. The spectrum exhibits a broad band in the range of 250–358 nm, which can be attributed to the ligand-centred S_0_ →S_1_ (π → π^*^) transition of the Hbtec^3–^ anion. The absorption bands indicate that the Hbtec^3-^ ligand has a higher triplet state energy level than Eu^3+^ and that energy can be efficiently transferred from the ligand to Eu^3+^. This result indicates that the excitation system can be sensitized by the Hbtec^3−^ ligand compared with Eu^3+^ absorption. This complex can be used for labelling gunshot residue (GSR), with useful applications in forensic detection and public safety (see Table 1 below).

Zhang et al. [62] prepared a EuL_n_-Fm/PVA hydrogel (Complex **78**) consisting of Eu(Sal)_3_ (Sal = salicylic acid) and a Eu(TTA)_3_ complex (L = Sal and TTA, Fm = formamide, n = 1–3), which enabled the continuous detection of ammonia. In this system, the Sal fluorescence burst due to its limitation in the Eu(Sal)_3_ complex and then recovered in the presence of ammonia. By introducing Eu(TTA)_3_ into the hydrogel, a binary system (EuLn-Fm/PVA hydrogel) was realized for the continuous detection of ammonia. According to the different emission spectra obtained at 421 nm, the EuL_n_-Fm/PVA hydrogel rapidly luminesces and undergoes a fluorescence burst from approximately 0–30 min under the stimulation of 5 mmol ammonia at room temperature (approximately 30 °C). After 30 min, the fluorescence gradually recovers in the ammonia environment. The coordination of Eu^3+^ and Sal with ammonia molecules in the hydrogel environment influences energy transfer from the ligands to the rare earth ions.

Li et al. [63] prepared four europium(III) ternary rare earth complexes (Complexes **79**–**82**) via a precipitation reaction using Eu_2_O_3_ as the raw material; anhydrous ethanol as the solvent system; methylene benzoyl (DBM), TTA, 1-(4-tert-butylphenyl)-3-(4-methoxyphenyl)−1,3-propanedione (avobenzone) or 2,4,6-trimethylbenzoic acid (TMBA) as the primary ligand; and TPPO as the secondary ligand. The effects of different primary ligands on the fluorescence intensity, fluorescence quantum efficiency, colour temperature, colour coordinates and thermal stability of the complexes were analysed from the perspective of energy transfer to explore the influence of the primary ligand on the fluorescence properties of the europium complexes. The excitation spectra showed that the maximum excitation wavelengths of Eu(TTA)_3_(TPPO), Eu(avobenzone)_3_(TPPO) and Eu(DBM)_3_(TPPO) are all approximately 395 nm, whereas the maximum excitation wavelength of Eu(TMBA)_3_(TPPO) is 375 nm. The maximum excitation wavelengths of the europium complexes are predominantly affected by the primary ligand TMBA. The results show that Eu(TTA)_3_TPPO and Eu(DBM)_3_(TPPO) have high quantum efficiencies of 93% and 85.9%, respectively, and that Eu(avobenzone)_3_(TPPO) and Eu(TMBA)_3_(TPPO) have a low quantum efficiency and low luminescence efficiency of 35% and 10%, respectively. Therefore, Eu(TTA)_3_TPPO has the strongest fluorescence, and Eu(TMBA)_3_(TPPO) has the weakest fluorescence. Eu(TTA)_3_TPPO is a red luminescent material with a wide range of applications.

Xia and others [64] combined sodium dicyanamide (Na-dca), pyrimidine-2-carboxylic acid (Hpmc) and europium nitrate under certain conditions to yield the coordination compounds [Eu(pmc)_2_(dca)(H_2_O)_2_]_n_ (Complex **83**). The chain structure of pyrimidine-2-carboxylic acid (Hpmc) was transformed into a two-dimensional net structure by O_water_-H..... O_carboxylate_ and O_water_-H·N_pyrimidine_ hydrogen bonding, which was then extended into a three-dimensional supramolecular structure by O_water_-H-N_cyano_ hydrogen bonding. The complex exhibited the characteristic fluorescence of Eu^3+^.

The spectral luminescence of europium(III) complexes with o-methylbenzoic acid and m-methylbenzoic acid was experimentally investigated by Kalinovskaya et al. [65]. The results showed that the luminescence intensity of europium(III) o-methylbenzoate is greater than that of m-europium(III)-methylbenzoate.

**Figure 28:**
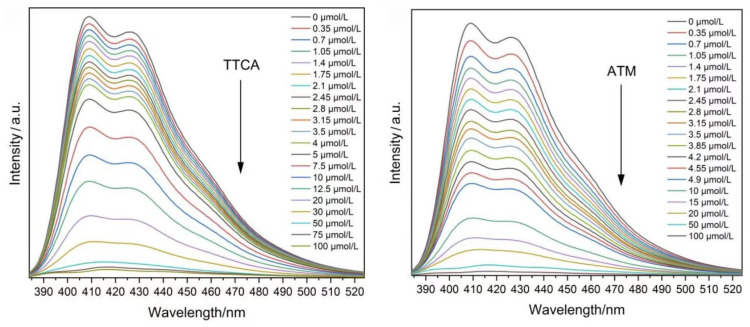
Emission spectra of Complex 84 after adding different concentrations of TTCA (λ_ex_ = 374 nm) and ATM (λ_ex_ = 374 nm) [66].

Tan et al. [66] used the anthracene-based ligand 5,5′-(anthracene-9,10-diyl)diisophthalic acid (H_4_adip) as a raw material to synthesize a luminescent lanthanide-organic framework [Eu_2_(adip)(H_2_adip)(DMF)_2_]-CH_3_OH (Complex **84**) via a solvothermal method. The fluorescence intensities of the produced complexes show excellent linear increases in the pH range of 4.8 to 7.1. The fluorescence enhancement reaches 588% per unit increase in pH, which is the highest value for fluorescent pH-sensing materials, thus improving the pH sensitivity in the detection range. In addition, the complexes can specifically recognize 2-thiothiazolidine-4-carboxylic acid (TTCA) and the antibiotic aztreonam (ATM) based on fluorescence bursts. In a few cases, Complex 84 showed the highest sensitivity to TTCA and ATM. The authors also investigated the pH, TTCA, and ATM sensing mechanisms. After the addition of 100 μmol·L^–1^ TTCA and ATM, there was almost no change in pH; therefore, the fluorescence burst was not induced by pH changes. Additionally, there was almost no change in the fluorescence lifetime, which confirmed that the burst process was static. The absorption peak shifted from 374 nm to 390 nm after the addition of TTCA and from 374 nm to 391 nm after the addition of ATM. Figure 28 [66] shows the emission spectra of Complex 84 after adding different concentrations of TTCA and ATM. With the addition of TTCA and ATM, the fluorescence of Complex **84** was gradually quenched. This quenching is caused by fluorescence resonance energy transfer (FRET). Complex **84** has a strong emission range of 384–524 nm, while TTCA and ATM have little absorption above 325 nm, so there is no overlap between the spectral peaks. In conclusion, the fluorescence burst of the complexes induced by TTCA and ATM can be attributed to the static burst caused by supramolecular interactions. The authors successfully constructed a pH sensor using H_4_adip for the first time, which provides a new idea for the design of pH sensors based on metal–organic frameworks (see Table 1 below).

**Figure 29:**
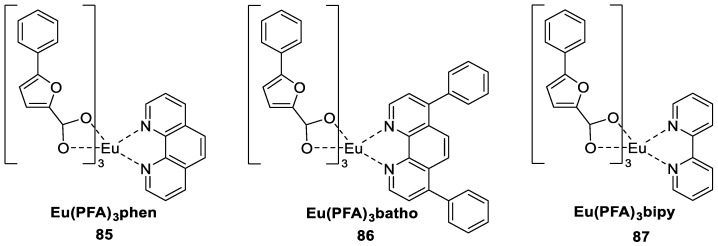
Chemical structures of **85**–**87**.

Bedi et al. [67] successfully synthesized a class of binary and ternary complexes by using 5-phenyl-2-furoic acid (PFA) as the primary ligand and 2,2′-bipyridyl (bipy), bathophenanthroline (batho) and 1,10-phenanthroline (phen) as secondary ligands (Complexes **85**–**87**) (Figure 29). All four complexes exhibit the characteristic red emission peak of Eu^3+^ at 613 nm under an excitation wavelength of 330 nm. Based on elemental analysis, TG analysis and Fourier transform infrared (FT-IR) spectroscopy, the carboxylic acid groups in the main chain of the ligands were predicted to be responsible for complexation between the ligands and metals. Among the complexes studied, the ternary complexes exhibited potential photoluminescence properties, with 12–21% higher quantum efficiencies than those of the binary complexes. This result indicates that the ligand has a significant sensitizing effect on the central europium ion, which is attributed to the fact that the auxiliary ligand acts as a support for the organic acid ligand during the sensitization process. The introduction of the auxiliary ligand reduces the nonradiative energy loss and enhances the luminescence (see Table 1 below).

**Figure 30:**
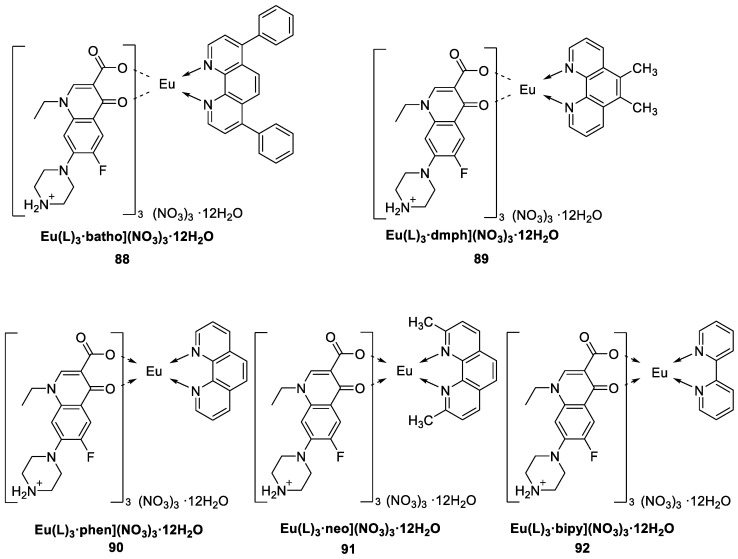
Chemical structures of **88**–**92**.

Ahlawat and coworkers [68] synthesized Eu^3+^ complexes via a grinding method with the primary ligand 1-ethyl-6-fluoro-1,4-dihydroxy-4-oxo-7-(1-piperazinyl)-3-quinoline carboxylic acid (L) and the secondary ligands 1,10-phenanthroline, bathophenanthroline, neocuproine, 2,2′-bipyridyl, and 5,6-dimethyl-1,10-phenanthroline (Complexes **88**–**92**) (Figure 30). The excitation spectra of the complexes include a broad band corresponding to the π^*^ → π electron transition of the ligands in the range of 200–550 nm, which is attributed to effective energy transfer from the ligand to the Eu^3+^ metal ion. Fluorine affects the thermal stability of the complexes and prolongs the luminescence lifetime. Among the five complexes, Complex **91** has the highest quantum yield of 41.91%, whereas the longest fluorescence lifetime is obtained for Complex **92** at 2.81 ms. An examination of the TG data reveals that these complexes have excellent thermal stability at 271 °C and can be applied as luminescent materials in OLED fabrication. Energy is transferred from the triplet state energy level of the ligand to the emission energy level of the europium(III) ion. Analysis of the biological properties of the complexes revealed that they had good antimicrobial and antioxidant effects (see Table 1 below).

**Figure 31:**
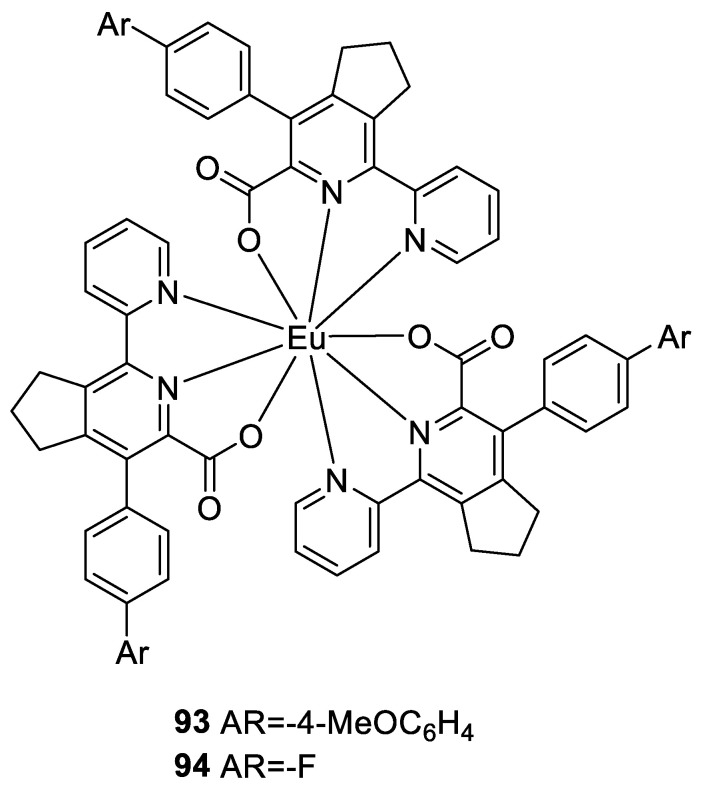
Chemical structures of **93** and **94**.

Krinochkin et al. [69] employed the “1,2,4-triazine” method, in which the intermediate 5-(4-bromophenyl)-2,2′-bipyridine-6-carbonitrile was subjected to Suzuki or Stille cross-coupling reactions to synthesize 5-(4-(Het)arylphenyl)-2,2′-bipyridine-6-carboxylic acids as neutral ligands. These ligands resulted in neutral europium complexes at a 3:1 stoichiometric ratio (Figure 31), and the photophysical properties of the complexes were investigated. In some cases, by introducing an aromatic ring into the 5-aryl substituent structure of the parent 2,2′-bipyridine ligand/chromophore, the photophysical properties of the lanthanide complexes improved. The highest quantum yield of 10.5% was obtained when the Ar group was 4-MeOCH_64_ (Complex **93**), and this complex also presented the longest fluorescence lifetime of 1.88 ms. In contrast, when the ligand was obtained without introducing an aromatic ring and -F was introduced into the ligand (Complex **94**), the quantum yield was the lowest at 4.2%, and the fluorescence lifetime was 0.95 ms (see Table 1 below).

Tyrosinase (TYR) is a copper-containing oxidase used as a reliable biomarker for melanoma and vitiligo. Dong and coworkers [70] synthesized three luminescent lanthanide materials: a dipicolinic acid and europium complex (DPA-Eu) (Complex **95**), a DPA and terbium complex (DPA-Tb) (Complex **96**), and a DPA-Eu/Tb (Complex **97**), with red, green, and yellowish fluorescence, respectively. These materials exhibit characteristic fluorescence reactions; the absorbance of the lanthanide sensing system at 420 nm gradually increased with increasing amounts of TYR. Under irradiation with 275 nm UV light, the red fluorescence of the solution decreased with increasing amounts of TYR, and when TYR was added to DPA-Eu, the fluorescence lifetime changed from the original 2.27 ns to 1.29 ns. Colorimetric analysis of the TYR activity was performed; thus, a lanthanide luminescent sensor for TYR activity was constructed from the above substances.

Naren et al. [71] synthesized a series of acyl amino acid europium complexes, Eu[CH_3_(CH_2_CH_2_)nCONHCH(CH_3_)COO]_3_ (n = 1~5) (Complexes **98**–**102**), using *N*-acyl amido alanine as a ligand and investigated their structures and photophysical properties. By comparing the luminescence intensity of the complexes with that of EuCl_3_, the acyl amino acid ligand was demonstrated to sensitize the characteristic luminescence of Eu^3+^. In addition, the effect of the solution concentration on the fluorescence properties was investigated; the fluorescence lifetime of Eu(ac-ala)_3_ increased when the concentration was increased within the range of 0.002–0.012 mol/L. The complexes aggregated at 0.014 mol/L, resulting in a decrease in their fluorescence lifetime. This behaviour implies that the longer the carbon chain is, the less likely the complexes are to aggregate in solution (the more hydrophobic they are, the stronger the repulsive force), and the higher the fluorescence burst concentration is. Finally, variable-temperature spectroscopy analysis elucidated the changes in the luminescence properties of the complexes with temperature. The fluorescence properties of complexes with different carbon chain lengths tend to change with temperature, and long-carbon-chain complexes have better high-temperature fluorescence stability. The authors investigated the luminescence intensity at temperatures from 25–160 °C, and the observed trends indicated that the six complexes can be categorized into three groups: (1) Eu(ac-ala)_3_, which shows a trend of decreasing luminescence intensity followed by increasing and then decreasing luminescence intensity; (2) Eu(oct-ala)_3_ and Eu(dec-ala)_3_, which exhibit an increase and then decrease in luminescence intensity; and (3) Eu(but-ala)_3_, Eu(hex-ala)_3_ and Eu(dod-ala)_3_ whose luminescence intensities decrease, stabilize, and then decrease again. The luminescence intensities of the coordinators change only slightly in the low-temperature region (20~60 °C) (see Table 1 below).

Polyurethane–europium materials prepared by introducing europium complexes into polyurethane have good luminescence properties. Li and others [72] synthesized a europium complex (Complex **103**) with double bonds using crotonic acid as the ligand and europium ions as the central ions. Then, the obtained europium complex was added to a synthesized polyurethane–acrylate monomer, and a polyurethane–europium material was prepared via polymerization with the polyurethane–acrylate monomer through the double bond in the complex. The prepared polyurethane–europium material has high transparency, good thermal stability, and good fluorescence; is amorphous; produces an absorption peak in the UV–visible spectrum below 400 nm; and exhibits high transmittance (more than 75%) under white light. With increasing amounts of the europium complex, the fluorescence intensity of the polyurethane–europium material consistently increases. There was no increase in fluorescence at europium complex concentrations from 0.5 wt% to 3 wt% because the europium complex bound to the polyurethane to a certain extent, preventing polyurethane aggregation and increasing the stability of the material. Since the europium complex content has opposite effects on the transmittance and fluorescence intensity of the material, the amount of the europium complex should be comprehensively considered when the final product is used as a transparent optical material. The fluorescence lifetime of the polyurethane–europium material (0.921 ms) is slightly longer than that of the pure europium complex (0.780 ms). This compound has potential application as a photoluminescent material, especially in high-transmittance applications.

Light conversion films are photoluminescent polymer thin-film materials [73]. They can convert light from one wavelength to another to realize wavelength control and can be widely used in liquid crystal displays, agricultural production, fluorescence detection, anticounterfeiting and other fields. Chen et al. [74] reported the synthesis and characterization of four europium(III) complexes with different carboxylic acid ligands, i.e., 2-pyridine-acrylic acid (H2-PA), 2-pyridine-carboxylic acid (HPic), benzoic acid (HBen), and cinnamic acid (HCin), as well as dinuclear europium–lanthanum complexes with HCin as the sole ligand (Complexes **104**–**108**). HBen and HPic were systematically investigated; and the excessively high triplet state energy levels of the Eu-Ben complex result in poor luminescence properties (quantum yield = 8.23%). Through ligand modification, such as introducing an electron-withdrawing group on the pyridine N atom at the para position of HPic, a ligand that better matches the lowest emission energy level of the europium ion can be obtained, which improves the photostability of the emitter. The coordination compounds rank from smallest to largest in terms of quantum yield as follows: Eu-2-PA < Eu-Ben < Eu-Pic < Eu-Cin < Ln-Eu-Cin. Comparative analysis of the performance of a light-converting agent and light-converting film before and after doping with La^3+^ revealed that doping with La^3+^ can not only increase the luminescence intensity of the light-converting agent and light-converting film but also increase the resistance of the light-converting film to UV ageing. Despite generally exhibiting good fluorescence stability, europium complexes may exhibit compromised fluorescence performance under certain extreme conditions, such as high temperature, high humidity, and intense light exposure, leading to weakened or diminished fluorescence; this can influence the authenticity verification performance, making it difficult to identify anticounterfeiting marks after long-term storage or exposure to harsh environments. Further improvement in this area is needed in the future (see Table 1 below).

Based on the literature, Eu^3+^ can be highly sensitized by π-electron-rich ligands. The introduction of phenyl, naphthyl, and other groups with large conjugated π-electron systems can increase the ligand light absorption ability and increase the energy transfer efficiency from the ligand to the europium ions. Auxiliary ligands act as supports for the primary ligand, and the introduction of auxiliary ligands generally reduces the nonradiative energy loss and enhances luminescence; in contrast, the introduction of neutral ligands results in an increase in the luminescence intensity. The introduction of heteroatoms, such as N, O, and S, can increase electron mobility and the triplet state energy, whereas the introduction of -F alone affects the thermal stability of the complex and generally increases the fluorescence lifetime of the complex. In addition, ligands with greater symmetry reduce the degree of distortion of the complex, which makes energy transfer more efficient and thus enhances the luminescence intensity; moreover, a rigid ligand structure helps maintain the geometrical configuration of the complex, reduces the chance of nonradiative transitions, and improves the luminescence efficiency.

## 5. Problems and Prospects

Through an extensive literature review, we learned that REEs are an asset to chemists. REEs must be fully integrated with nanomaterials to better synthesize REE-based electroluminescent materials, especially for OLEDs and solar energy conversion, with higher optical quality and easily adjustable refractive indices and emission colours. The life sciences will also see rapid advances in luminescent bioprobes based on REEs, but more efficient near-infrared-emitting probes are needed to increase the depth of penetration for imaging.

The combination of europium complexes and nanoparticles has been a focus of fluorescent probe research in recent years. Fluorescent probes with europium complexes have the advantages of sensitive detection, reusability and ease of use in the molecular probing of biological systems. Fluorescent probes can be used in combination with signal amplification techniques, but such methods can usually only detect a fixed target substrate and cannot be used to study complex and variable life systems; additionally, their application in the clinic is still very limited.

To date, europium complexes have been introduced into polymers, porous materials, and layered materials to form nanohybridized materials with improved luminescent properties [75,76,77]. Scalable clay minerals, graphite, and other substances are characterized by interlayer spaces, one of the most prominent features of layered materials [78]. Hybrid luminescent materials can be prepared by using clay minerals as carrier substrates of lanthanide complexes, which is not only inexpensive and simple but also green and environmentally harmless.

Rare earth complexes have been a significant research focus in the field of electroluminescence in recent decades. The innovative application of rare earth complexes as sensitizers in OLEDs allows the advantages of rare earth complexes and transition metal complexes to complement each other, thus significantly improving the luminescence performance of OLEDs. With the rapid development of science and technology, and to meet future needs related to 8 k TV and telemedicine, ultrahigh-definition imaging with high colour saturation has become a critical goal in OLED development.

In the field of photovoltaic power generation, silicon-based solar cells occupy a large portion of the market but still face the problem of low photoelectric conversion efficiency, which is partly attributed to their low ultraviolet light utilization efficiency. Europium complexes can convert UV light into visible light, which is expected to improve the photoelectric conversion efficiency of silicon-based solar cells [79]. However, the low stability of these complexes limits their practical application. Enhancing the absorbance and stability of rare earth complexes through molecular design is a complex yet challenging task that is predicated on a profound understanding of the interactions between rare earth ions and ligands. Modifying the structure of ligands, such as by introducing flexible groups or electron-transport groups, can alter the electron cloud distribution of the complexes, thereby affecting their absorbance. Additionally, through molecular design, the spatial structure of the complexes can be adjusted to make them more compact and stable. For example, the formation of stable structures such as six-membered chelating rings helps resist the impact of external factors on complex stability. Finally, the stability of the complexes can be further increased by incorporating protective groups into the ligands, such as hydroxyl or amino groups, which can form additional interactions with rare earth ions. These approaches can, to a certain extent, increase the absorbance and stability of rare earth complexes.

Eu^3+^ complexes have problems such as low quantum yield and thermal instability. To meet the needs of practical applications, the quantum yield of these complexes must be increased to over 80%. To realize thermal stability, the decomposition temperature of Eu^3+^ complexes needs to be increased to above 400 °C. For instance, encapsulation in ionic liquid matrices (e.g., [BMIM][PF₆]) elevates the decomposition temperature, and MOF confinement prevents oxidative/hydrolytic degradation at high temperatures. Although Eu^3+^ complexes have certain problems, their monochromatic red emission and broad spectral band make them irreplaceable among red light emitters. Future work will further improve these complexes to increase their activity and stability and optimize intramolecular energy transfer to promote the emission of more stable and higher-purity red light.

Despite significant advancements in Eu^3^⁺ β-diketonate/carboxylate complexes for luminescent applications, critical challenges and opportunities persist. Niobate hosts such as potassium sodium niobate (KNN) exhibit unique potential due to their low-symmetry perovskite structures, which enhance electric dipole transitions and enable dual ferroelectric-luminescence functionalities. However, practical adoption is hindered by the high synthesis temperature, limited Eu^3^⁺ doping concentration, and poor stability under humid conditions. Future efforts should prioritize low-temperature synthesis routes to minimize defects in niobates in conjunction with machine learning-guided design of ligands (e.g., fluorinated β-diketones) to optimize polarizability and thermal stability.

In summary, REE luminescence has important applications in almost every area of our society today. In the future, such luminescence could help solve two major technological challenges, i.e., how to provide medical assistance and sufficient sustainable energy for a rapidly growing population, so there is a need for continued research on rare earth luminescent materials.

**Table 1:** Photophysical properties of europium complexes of β-diketones and organic carboxylic acids.

Complex	λ_max, abs_ (nm)	λ_max, PL_ (nm)	Φ_em_ (%)	τ (ms)	Reference
**1**		614	41	0.55	[35]
**2**		614	40	0.51	[35]
**3**	279, 348	614	54		[36]
**4**	279, 348	614	52		[36]
**5**	279, 348	614	52		[36]
**6**	279, 348	614	54		[36]
**7**	279, 348	614	56		[36]
**8**	264–310, 317–368	624	2	0.80	[37]
**9**	265–295, 312–364, 392–406	615	7	0.65	[37]
**11**	385	616	20	0.43	[40]
**12**	370	616	14	0.37	[40]
**13**	286–292	539, 579, 596, 619, 652	13	0.52	[41]
**14**	286–292	539, 579, 596, 619, 652	24	0.96	[41]
**15**	286–292	539, 579, 596, 619, 652	28	1.09	[41]
**16**	286–292	539, 579, 596, 619, 652	39	1.52	[41]
**17**	286–292	539, 579, 596, 619, 652	46	1.77	[41]
**18**	300	582, 595, 616, 654, 700	46	2.47	[42]
**19**	300	582, 595, 616, 654, 700	51	2.56	[42]
**20**	300	582, 595, 616, 654, 700	51	2.72	[42]
**21**	300	582, 595, 616, 654, 700	50	2.82	[42]
**22**	300	582, 595, 616, 654, 700	51	2.95	[42]
**23**	300	582, 595, 616, 654, 700	54	3.08	[42]
**24**	352	580, 592, 611, 649, 700	14	1.32	[43]
**25**	352	580, 592, 611, 649, 700	19	0.34	[43]
**26**	352	580, 592, 611, 649, 700	25	0.56	[43]
**27**	352	580, 592, 611, 649, 700	28	0.67	[43]
**28**	352	580, 592, 611, 649, 700	33	0.64	[43]
**29**	274	614	41	0.66	[44]
**30**	286	614	44	0.75	[44]
**31**	290	614	49	0.84	[44]
**32**		614	38	0.35	[45]
**33**		613	41	0.38	[45]
**34**		614	52	0.43	[45]
**35**	289	615	0.01		[46]
**36**	324	615	0.8	0.69	[46]
**37**	352	616	0.3		[46]
**38**	362	615	0.007		[46]
**39**	368	615			[46]
**40**	255	615			[46]
**41**				0.33	[47]
**42**				0.79	[47]
**43**	280–450	578, 592, 613, 650, 702	4		[48]
**44**	295	615	78		[50]
**45**	295	616	57		[50]
**46**	295	614	82		[50]
**47**	295	618	78		[50]
**48**	295	616	30		[50]
**49**	250, 345	614	6		[50]
**50**	250, 345	614	7		[50]
**51**	250, 345	615	10		[50]
**52**	250, 345	621	5		[50]
**53**	250, 345	617	4		[50]
**54**	330, 390	610		0.39	[21]
**55**	337	611	11	0.98	[51]
**56**	340	612	7	0.69	[51]
**57**	341	613	5	0.62	[51]
**58**	344	611	4	0.43	[51]
**59**	349	613, 617		0.84	[52]
**60**	350	612, 614		0.53	[52]
**61**	352	612, 614		0.49	[52]
**62**	353	612, 614		0.48	[52]
**63**	260–380	578, 592, 614, 650, 702	22	0.46	[53]
**64**	260–380	578, 592, 614, 650, 702	16	0.55	[53]
**65**	260–380	578, 592, 614, 650, 702	11	0.37	[53]
**66**		600–625	7		[54]
**67**		600–625	7		[54]
**68**	275	617			[55]
**69**		616		0.42	[56]
**70**		580, 592, 616, 653, 700		0.53	[56]
**71**	273	615, 657, 698	28	1.27	[57]
**72**	273	615, 661, 698	36	1.53	[57]
**73**	282	615, 657, 699	37	1.57	[57]
**74**		615		2.30	[58]
**75**	240, 283	592, 616, 696	36		[59]
**76**	330	580, 593, 617, 651, 700	28	0.77	[60]
**77**		614	37	1.22	[61]
**84**	374	409		5.51 × 10^−6^	[66]
**85**	302	580, 592, 613	13	0.53	[67]
**86**	302	580, 592, 613	15	0.60	[67]
**87**	302	580, 592, 613	22	0.83	[67]
**88**	281, 322	582, 595, 615, 654, 700	27	2.52	[68]
**89**	281, 322	582, 595, 615, 654, 700	32	2.68	[68]
**90**	281, 322	582, 595, 615, 654, 700	32	2.61	[68]
**91**	281, 322	582, 595, 615, 654, 700	42	2.70	[68]
**92**	281, 322	582, 595, 615, 654, 700	41	2.81	[68]
**93**	231, 271	592, 615, 651, 696	11	1.88	[69]
**94**	232, 294	592, 615, 651, 696	4	0.95	[69]
**98**	219, 241	590, 610, 650, 698	20	0.60	[71]
**99**	219, 241	590, 610, 650, 698	23	0.70	[71]
**100**	219, 241	590, 610, 650, 698	16	0.40	[71]
**101**	219, 241	590, 610, 650, 698	29	0.70	[71]
**102**	219, 241	590, 610, 650, 698	25	0.60	[71]
**104**		592, 615	1	0.25	[74]
**105**		592, 615	13	1.49	[74]
**106**		592, 615	8	0.39	[74]
**107**		592, 615	22	0.58	[74]
**108**		592, 615	33	0.84	[74]

## Data Availability

No new data were created or analyzed in this study. Data sharing is not applicable to this article.
